# Impact of Difluoromethylornithine and AMXT 1501 on Gene Expression and Capsule Regulation in *Streptococcus pneumoniae*

**DOI:** 10.3390/biom14020178

**Published:** 2024-02-02

**Authors:** Moses B. Ayoola, Leslie A. Shack, Otto Phanstiel, Bindu Nanduri

**Affiliations:** 1 Department of Comparative Biomedical Sciences, College of Veterinary Medicine, Mississippi State University, Mississippi State, MS 39762, USA; mba185@msstate.edu (M.B.A.); shack@cvm.msstate.edu (L.A.S.); 2 Department of Medical Education, College of Medicine, University of Central Florida, Orlando, FL 32826, USA; otto.phanstiel@ucf.edu

**Keywords:** *Streptococcus pneumoniae*, polyamine, DFMO, AMXT 1501, capsule

## Abstract

*Streptococcus pneumoniae* (Spn), a Gram-positive bacterium, poses a significant threat to human health, causing mild respiratory infections to severe invasive conditions. Despite the availability of vaccines, challenges persist due to serotype replacement and antibiotic resistance, emphasizing the need for alternative therapeutic strategies. This study explores the intriguing role of polyamines, ubiquitous, small organic cations, in modulating virulence factors, especially the capsule, a crucial determinant of Spn’s pathogenicity. Using chemical inhibitors, difluoromethylornithine (DFMO) and AMXT 1501, this research unveils distinct regulatory effects on the gene expression of the Spn D39 serotype in response to altered polyamine homeostasis. DFMO inhibits polyamine biosynthesis, disrupting pathways associated with glucose import and the interconversion of sugars. In contrast, AMXT 1501, targeting polyamine transport, enhances the expression of polyamine and glucose biosynthesis genes, presenting a novel avenue for regulating the capsule independent of glucose availability. Despite ample glucose availability, AMXT 1501 treatment downregulates the glycolytic pathway, fatty acid synthesis, and ATP synthase, crucial for energy production, while upregulating two-component systems responsible for stress management. This suggests a potential shutdown of energy production and capsule biosynthesis, redirecting resources towards stress management. Following DFMO and AMXT 1501 treatments, countermeasures, such as upregulation of stress response genes and ribosomal protein, were observed but appear to be insufficient to overcome the deleterious effects on capsule production. This study highlights the complexity of polyamine-mediated regulation in *S. pneumoniae*, particularly capsule biosynthesis. Our findings offer valuable insights into potential therapeutic targets for modulating capsules in a polyamine-dependent manner, a promising avenue for intervention against *S. pneumoniae* infections.

## 1. Introduction

*Streptococcus pneumoniae* (Spn), the causative agent of community-acquired pneumonia and invasive pneumococcal disease, is responsible for over 700,000 annual deaths of children under the age of five worldwide [1]. Spn is the predominant pathogen associated with pneumonia-related deaths on a global scale, contributing to approximately 1.2 million deaths annually across all age groups [2]. The introduction of pneumococcal conjugate vaccines and pneumococcal polysaccharide-based vaccines initially led to a reduction in infections caused by the vaccine-targeted serotypes. However, serotype replacement [3] and increasing antibiotic resistance among pneumococcal serotypes complicate treatment strategies [4].

Numerous alternatives to traditional vaccines and antibiotics have been proposed to treat pneumococcal infections. These include the use of probiotics, specifically the *Lactobacillus casei* strain [5], phage therapy [6,7], and a variety of non-capsular proteins, such as pneumococcal surface adhesin A (PspA), pneumococcal histidine triad protein D (PhtD), pneumococcal choline-binding protein A (PcpA), and pneumolysin (PLY) and its derivative (PlyD). Combinations of these proteins are also being explored for vaccine production [8]. However, these strategies are currently in the experimental and evaluation phases. In recent years, a set of promising novel proteins has been identified as potential candidates for preventing and treating pneumococcal infections. These proteins are conserved across multiple pneumococcal serotypes and are intricately connected to metabolic biosynthesis and transport pathways involving small biogenic molecules known as polyamines [9]. The deletion of PotD, which represents the substrate-binding domain of the polyamine transporter, has been demonstrated to be essential for the virulence of encapsulated pneumococci [10]. Immunization with a hybrid pneumolysin derivative and PotD has been found to generate a robust immune response [11]. Furthermore, studies involving recombinant PotD alone [12] or in combination with PspA [13] have shown promise in reducing colonization in mouse models.

Polyamines are a group of organic compounds that play essential roles in various biological processes. They are characterized by multiple amino (NH_2_) groups and are typically found as small organic cations at physiological pH. The most common polyamines in biological systems are cadaverine, putrescine, spermidine, and spermine [14]. Polyamines are multifaceted compounds that can act as antioxidants, shielding cells from oxidative stress while simultaneously orchestrating the meticulous regulation of cell growth and proliferation [15]. Their dynamic interplay with DNA, RNA, and proteins enables polyamines to regulate gene expression, influencing complex and well-coordinated activities taking place in living systems [16].

We previously demonstrated that the deletion of a polyamine biosynthesis gene, *SP_0916*, arginine decarboxylase, in pneumococcal serotype 4 (TIGR4) resulted in a reduction of the capsule [17], the predominant virulence factor. Deletion of *SP_0916* also led to the downregulation of proteins associated with polyamine synthesis and cell wall synthesis. Interestingly, there was an upregulation of proteins from the pentose phosphate pathway (PPP), indicating an enhanced oxidative stress response. A similar shift in pneumococcal metabolism that favors PPP was noted with the gene expression profile of *SP_0916* [18]. Metabolomics studies support the notion that polyamines could exert control over capsule synthesis at the transcriptional level [18]. Deletion of the polyamines transport operon *(∆potABCD*) resulted in a noticeable decrease in intracellular polyamine levels [19] and an increase in susceptibility to oxidative and nitrosative stress [20]. Similar to the *SP_0916* deletion, there was an upregulation in the expression of genes associated with the PPP and the arginine deiminase system as an adaptive mechanism to manage stress conditions in *∆potABCD* [20]. Chemical inhibition of polyamine biosynthesis with difluoromethylornithine (DFMO), an irreversible inhibitor of eukaryotic ornithine decarboxylase (ODC), as an alternative and complementary approach to validate the observations made through genetic manipulation indicated loss of capsule in multiple pneumococcal serotypes [21]. However, capsule inhibition was noted only in serotype 2 (D39) when treated with AMXT 1501, an inhibitor of polyamine transport.

As reported in the previous study [21], DFMO and AMXT 1501 treatment resulted in reduced levels of agmatine, *N*-carbamoylputrescine, putrescine, and cadaverine. Reduced levels of spermine were observed with DFMO, while AMXT 1501 resulted in reduced levels of spermidine and *N*-acetylspermidine [21]. In this study, we further characterized gene expression changes in D39 in response to DFMO and AMXT 1501 to identify mechanisms that could explain its susceptibility to these compounds that result in reduced capsule. Our findings indicate that DFMO and AMXT 1501 regulate capsule biosynthesis by targeting precursor availability and ATP, respectively.

## 2. Materials and Methods

### 2.1. Bacterial Growth and RNA Isolation

Total RNA was extracted and purified from mid-log phase cultures (optical density at 600 nm between 0.4 and 0.5) of *Streptococcus pneumoniae* D39, grown in THY medium (*n* = 4), both in the absence and presence of DFMO and AMXT 1501. The compounds were added at non-lethal doses but at concentrations that significantly impaired capsule production, specifically at ⅛ MIC DFMO (34 mM) and ½ MIC AMXT 1501 (7.4 µM) [21]. RNA purification was carried out using the Rneasy Midi Kit (Qiagen, Valencia, CA, USA). The quality of the isolated RNA was assessed using an Agilent 2100 Bioanalyzer (Agilent Technologies, Wilmington, DE, USA). Purified total RNA was utilized for conducting RNA-Seq and quantitative RT-PCR experiments.

### 2.2. RNASeq and Data Analysis

For RNA-Seq analysis, libraries were prepared employing the KAPA RNA Hyper Kit with RiboErase (Kapa Biosystems, Wilmington, MA, USA), using 5 µg of RNA as input. The library preparation workflow included rRNA removal, cDNA synthesis, end repair to generate blunt ends, A-tailing, adaptor ligation, and PCR amplification. Various adaptors were employed to facilitate the multiplexing of samples for concurrent sequencing in a paired-end run. Library concentrations and quality were accurately determined using the Qubit ds DNA HS Assay Kit (Life Technologies, Carlsbad, CA, USA) and the Agilent Tapestation (Agilent Technologies, Wilmington, DE, USA).

Subsequent sequencing was carried out on an Illumina Hiseq 3000 platform, generating paired-read 150-base pair sequences. Data quality assessment was conducted using Illumina SAV version 2.4.7, and the de-multiplexing of sequences was carried out with Illumina Bcl2fastq2 version 2.17. In-depth analyses, including the removal of failed reads, mapping of the short sequence reads to the reference genome of Spn D39, and the identification of differentially expressed genes, were performed using the RNA-Seq tool within CLC Genomic Workbench 23.0.3 (Qiagen, Valencia, CA, USA). The mapping process used CLC’s proprietary read mapper with specific parameters, including a mismatch cost of 2, an insertion or deletion cost of 3, and a similarity/length fraction of 0.8. Read counting was achieved with the EM estimation algorithm, and differentially expressed genes were identified based on fold changes generated by a Generalized Linear Model approach akin to the edgeR algorithm. Genes with a false discovery rate (FDR) of ≤0.05 were considered statistically significant, and we used a fold change cut-off of ±1.3. RNA-Seq raw data and metadata reported in this study are available at NCBI GEO with the accession number GSE252370.

To gain deeper insights into the functional alterations within Spn D39 treated with DFMO and AMXT 1501, the differentially expressed genes were analyzed by integrating multiple bioinformatics resources such as KEGG [22] and UniProt [23]. These resources were used to infer the biological functions that were affected in response to the inhibition of polyamine synthesis and transport.

### 2.3. Quantitative Real-Time PCR (qRT-PCR)

We performed quantitative reverse transcription PCR (qRT-PCR) with selected genes to validate the gene expression changes detected by RNASeq. The primer sequences utilized for qRT-PCR are shown in Table 1. All primers were rigorously validated through melt curve analysis using SYBR Green (Thermo Fisher Scientific, Waltham, MA, USA). In brief, the purified RNA (7.5 ng per reaction) was reverse-transcribed into cDNA, and PCR amplification was performed using the SuperScript III Platinum SYBR Green One-Step qRT-PCR Kit (Thermo Fisher Scientific, Waltham, MA, USA). All analyses were conducted using three biological replicates and three technical replicates for each experiment. The relative quantification of gene expression was determined using the Stratagene Mx3005P qPCR System (Agilent Technologies, Santa Clara, CA, USA). The expression of the selected genes was normalized to the expression of *gapdh*, and fold changes were calculated using the comparative CT method.

## 3. Results

### 3.1. Gene Expression Changes and Pathways Affected by DFMO in Pneumococcal D39

DFMO treatment resulted in significant changes in the expression of 96 genes, 49 of which were upregulated and 47 were downregulated in Spn D39 compared to the untreated samples (Supplementary Table S1). The following sections describe the genes/pathways regulated by DFMO.

#### 3.1.1. DFMO Negatively Impacts Polyamine Biosynthesis Pathways

**Figure 1:**
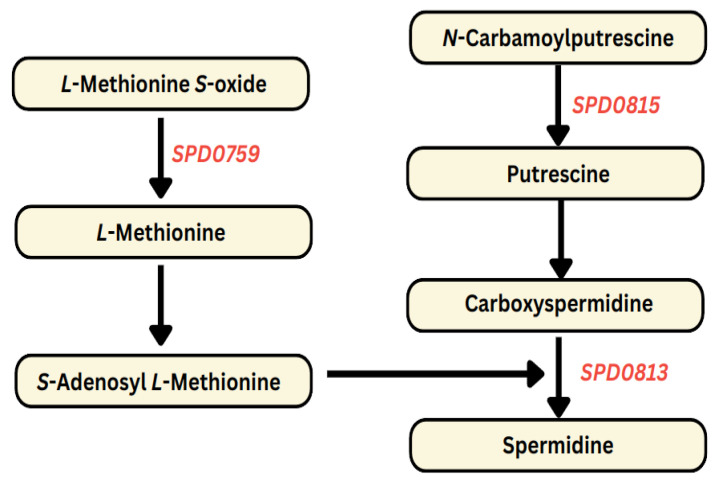
DFMO treatment results in the downregulation of putrescine and spermidine biosynthesis in Spn D39. Notably, *SPD0759*, *SPD0813*, and *SPD0815*, which encode a GAF domain-containing protein, carboxynorspermidine decarboxylase, and *N*-carbamoylputrescine amidase, respectively—all crucial components of polyamine biosynthesis pathways—are depicted as downregulated (italicized in red).

DFMO treatment resulted in the downregulation of genes from the putrescine and spermidine biosynthesis pathways: *SPD0815* (−1.5) encodes *N*-carbamoylputrescine amidase that catalyzes the hydrolysis of *N*-carbamoylputrescine to putrescine, and *SPD0813* (−1.5) (carboxynorspermidine decarboxylase) that catalyzes decarboxylation of carboxypermidine to spermidine (Figure 1). *SPD0759* (−1.5) encodes a GAF domain-containing protein that catalyzes the conversion of *L*-Methionine *S*-oxide to *L*-Methionine. The precursor of *S*-Adenosyl *L*-Methionine (SAM) that donates the aminopropyl moiety for the synthesis of spermidine is also downregulated. The implication of these findings is that DFMO, as a polyamine biosynthesis inhibitor, effectively regulates polyamine production at the transcriptional level. While DFMO is commonly recognized as an irreversible inhibitor of ornithine decarboxylase, earlier studies have demonstrated its capability to inhibit arginine decarboxylase, leading to the inhibition of agmatine production and subsequently *N*-carbamoylputrescine within the alternative polyamine biosynthesis pathway [21,24].

#### 3.1.2. DFMO Inhibits Expression of Genes Involved in Glucose Production

**Figure 2:**
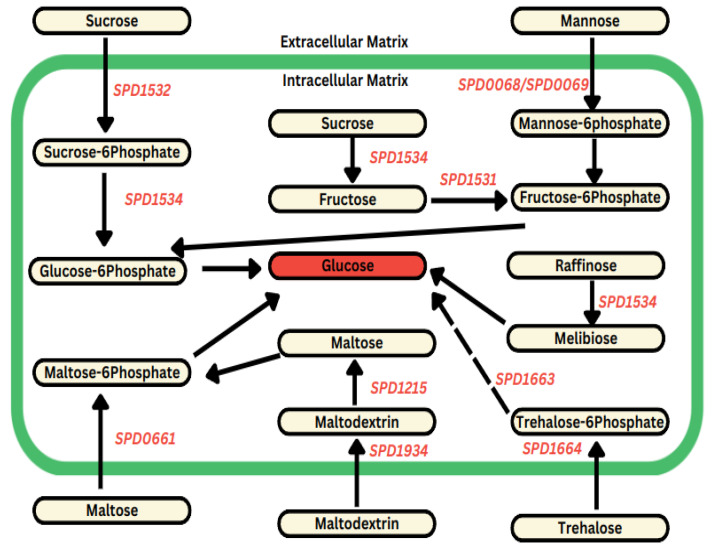
DFMO exerts negative effects on the expression of genes regulating glucose availability. Treatment of Spn D39 with DFMO results in the downregulation (shown in red) of genes associated with glucose accessibility. This includes the phosphotransferase system (*SPD1532*, *SPD0068*, *SPD0069*, *SPD1664*, and *SPD0661*) responsible for importing other hexose sugars besides glucose, oligosaccharide transport (*SPD1934*), as well as genes involved in starch and sucrose metabolism (*SPD1534*, *SPD1215*, *SPD1531*, and *SPD1663*) aimed at converting various substrates into glucose. Multi-step reactions are represented by a broken arrow.

The repeat unit sugars in the Spn D39 capsule consist of one glucuronic acid molecule, two glucose molecules, and three rhamnose molecules [25]. Interestingly, both rhamnose and glucuronic acids share glucose as a key biosynthetic precursor, underscoring the paramount role of glucose as the primary and essential metabolite in Spn D39 capsule biosynthesis [26,27]. In this study, we observed the downregulation of various genes encoding the phosphotransferase system (PTS), responsible for importing different simple sugars from the extracellular matrix into the intracellular environment. These sugars can then be converted into glucose. Notable significant downregulated genes include *SPD1532* (−2.5) (sucrose-specific PTS transporter subunit IIBC), *SPD0068* (−1.8) (PTS system mannose/fructose/sorbose family transporter subunit IID), *SPD0069* (−2.1) (PTS sugar transporter subunit IIA), *SPD1664* (−3.5) (PTS system trehalose-specific EIIBC component), *SPD0661* (−1.6) (PTS transporter subunit IIBC), and the oligosaccharide transporter *SPD1934* (−1.5) (maltodextrin ABC transporter substrate-binding protein) (Figure 2). Additionally, the expression of several genes encoding proteins responsible for catalyzing the interconversion of various sugars into glucose is downregulated. Genes such as *SPD1534* (−3.0) (sucrose-6-phosphate hydrolase), *SPD1215* (−2.0) (alpha-amylase), *SPD1531* (−2.1) (ROK family protein), and *SPD1663* (−3.6) (alpha, alpha-phosphotrehalase) are among those showing reduced expression (Figure 2). The qRT-PCR results are consistent with RNA-seq data, as there was a significant reduction in the expression of *SPD1663* (−5.2-fold) and *SPD1664* (−3.7-fold).

#### 3.1.3. DFMO Suppression of Phosphate Operon and Iron Importer Gene Expression

Our results identify a significant downregulation in the expression of genes related to phosphate operon transporters. Specifically, expression of genes such as *SPD1910* (−5.6) (encodes PstS, a substrate-binding domain-containing protein), *SPD1911* (−4.8) (encoding PstC, the phosphate ABC transporter permease subunit PstC), *SPD1912* (−5.1) (encodes PstA, the phosphate ABC transporter permease PstA), *SPD1913* (−4.9) (encodes pstB, the phosphate ABC transporter ATP-binding protein PstB), and *SPD1614* (−6.7) (encodes phosphate uptake regulator PhoU) that are associated with phosphate import. Additionally, we observed reduced expression of *SPD1607* (−2.7), which encodes the iron ABC transporter permease, indicating an impact on iron import as well. The upregulation of these phosphate transport proteins has previously been demonstrated to enhance the fitness of Spn and confer resistance to antibiotics [28,29]. Furthermore, iron is a well-known essential trace element crucial for virulence and stress response in Spn [30,31]. These observations align with earlier findings, supporting the notion that inhibiting polyamines not only hinders pneumococcal capsule synthesis but also increases vulnerability to stress.

### 3.2. Pneumococcal Genes and Pathways Affected by AMXT 1501

In the case of AMXT 1501-treated D39, a total of 544 genes were identified as significantly altered, comprising 281 upregulated and 263 downregulated genes (Supplementary Table S2). The subsequent sections provide details regarding the genes and pathways responsive to AMXT 1501.

#### 3.2.1. AMXT 1501 Enhances the Expression of Polyamine Biosynthesis

**Figure 3:**
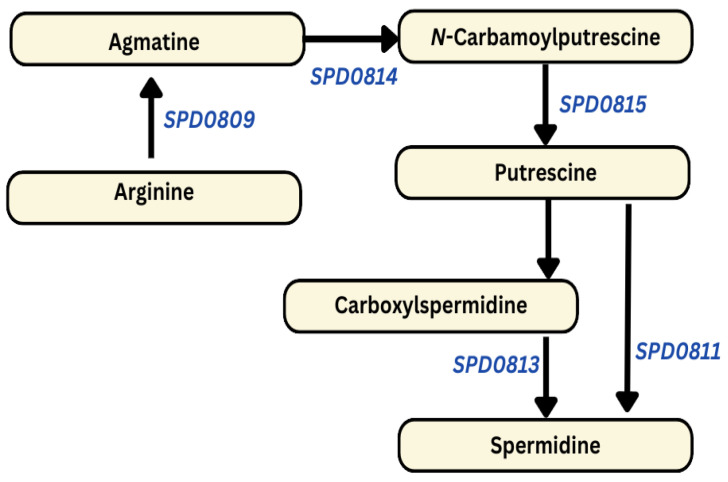
AMXT 1501 results in the upregulation of putrescine and spermidine biosynthesis in Spn D39. Specifically, *SPD0809*, *SPD0814*, *SPD0815*, *SPD0813*, and *SPD0811*, which encode aminotransferase class I/II-fold pyridoxal phosphate-dependent enzymes, agmatine deiminase, *N*-carbamoylputrescine amidase, carboxynorspermidine decarboxylase, and polyamine aminopropyltransferase, respectively—all crucial components of polyamine biosynthesis pathways—are depicted as upregulated (italicized in blue).

Inhibition of polyamine transport by AMXT 1501 upregulated the expression of polyamine biosynthesis genes, specifically *SPD0815* (1.4), *SPD0811* (1.7), and *SPD0813* (1.6) (Figure 3). These genes encode *N*-carbamoylputrescine amidase, polyamine aminopropyltransferase, and carboxynorspermidine decarboxylase, respectively, contributing to the synthesis of putrescine from *N*-carbamoylputrescine, followed by the synthesis of spermidine from putrescine and carboxylspermidine. Furthermore, the upregulation is observed in *SPD0809* (1.9) and *SPD0814* (1.5), which encode aminotransferase class I/II-fold pyridoxal phosphate-dependent enzymes and agmatine deiminase, respectively. These enzymes are involved in the synthesis of polyamine intermediates (agmatine and *N*-Carbamoylputrescine) from the precursor amino acid arginine. The qRT-PCR results are consistent with RNA-seq data, as there was a significant upregulation in the expression of *SPD0811* (3.3-fold), *SPD0813* (2.7-fold), and *SPD0815* (2.4-fold).

#### 3.2.2. AMXT 1501 Enhances the Expression of Genes Involved in Glucose Production

**Figure 4:**
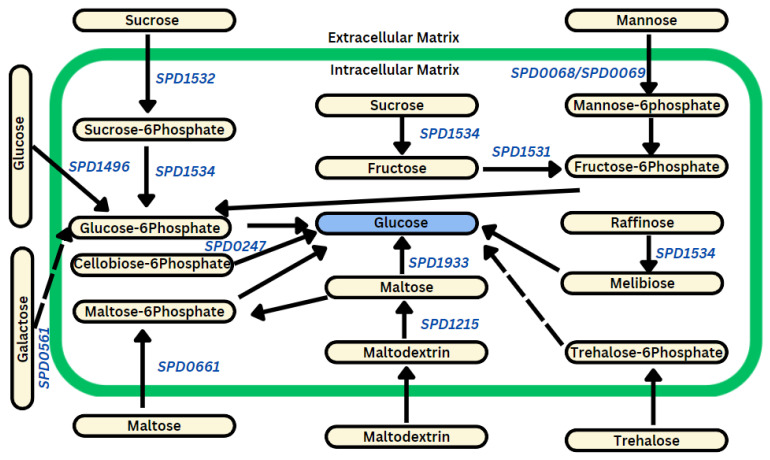
AMXT 1501 upregulates the expression of genes that control glucose availability. Treatment of Spn D39 with AMXT 1501 results in the upregulation (shown in blue) of genes associated with glucose availability. This includes the phosphotransferase system (*SPD1496*, *SPD0561*, *SPD1532*, *SPD0068*, *SPD0069*, and *SPD0661*) responsible for importing glucose and other hexose sugars, as well as genes involved in starch and sucrose metabolism (*SPD1534*, *SPD1215*, *SPD1531*, *SPD1933*, and *SPD0247*) aimed at converting various substrates into glucose. Multi-step reactions are represented by a broken arrow.

Following AMXT 1501 treatment, notable upregulation of multiple genes encoding phosphotransferase system (PTS), including *SPD1496* (2.2) (PTS transporter subunit EIIC), *SPD0561* (7.2) (PTS galactitol transporter subunit IIC), *SPD1532* (5.9) (sucrose-specific PTS transporter subunit IIBC), *SPD0068* (3.0) (PTS system mannose/fructose/sorbose family transporter subunit IID), *SPD0069* (3.4) (PTS sugar transporter subunit IIA), and *SPD0661* (1.4) (PTS transporter subunit IIBC) (Figure 4), was observed. Additionally, the expression of several genes involved in the conversion of various sugars into glucose as pneumococcal D39 capsule precursors, such as *SPD1534* (3.9) (sucrose-6-phosphate hydrolase), *SPD1215* (1.6) (alpha-amylase), *SPD1531* (2.8) (ROK family protein), *SPD1933* (4.7) (4-alpha-glucanotransferase), and *SPD0247* (2.3) (6-phospho-beta-glucosidase), was upregulated. The qRT-PCR results are consistent with RNA-seq data, as there was a significant upregulation in the expression of *SPD1496* (2.0-fold) and *SPD0561* (2.7-fold).

#### 3.2.3. AMXT 1501 Treatment Inhibits ATP Production

**Figure 5:**
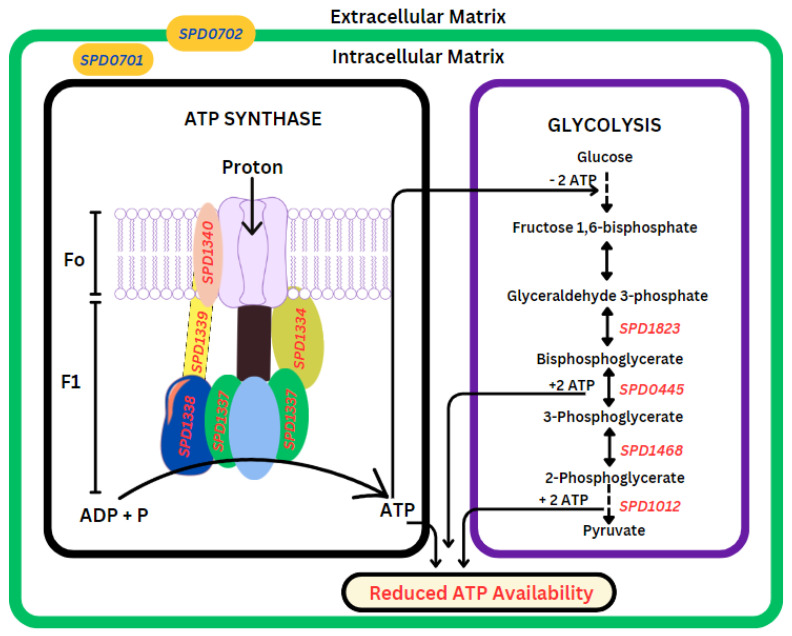
Impact of AMXT 1501 on ATP synthase and glycolysis. AMXT 1501 significantly inhibits adenosine triphosphate (ATP) production. This is manifested by a pronounced downregulation of genes (shown in red) associated with the ATP synthase system, including *SPD1334*, *SPD1337*, *SPD1338*, *SPD1339*, and *SPD1340*, which encode essential components of the F0F1 ATP synthase subunits. The ATP synthase system, responsible for generating ATP from the transmembrane proton gradient, experiences significant suppression. In the glycolytic pathway, key enzymes encoded by *SPD1823*, *SPD0445*, *SPD1468*, and *SPD1012*, involved in converting glucose to pyruvate, are notably downregulated. These enzymes play a crucial role in ATP production during glycolysis. Observed changes in gene expression appear to be under the regulation of the two-component regulatory system (CiaRH: *SPD0701* (3.7)/*SPD0702* (3.9)).

AMXT 1501 treatment led to a significant downregulation of genes involved in adenosine triphosphate (ATP) production. Expression of ATP synthase and glycolytic pathway genes was reduced (Figure 5). ATP synthase is an intricate molecular apparatus consisting of two multimeric subunits—the membrane-bound Fo subunit and the F1 subunit. This complex machinery is accountable for the synthesis of ATP from the transmembrane proton gradient. We noted significant downregulations in genes related to the ATP synthase system, including *SPD1334* (−1.6), *SPD1337* (−1.4), *SPD1338* (−1.6), *SPD1339* (−1.8), and *SPD1340* (−1.4). These genes encode crucial components: F0F1 ATP synthase subunits epsilon, alpha, delta, B, and A, respectively. The ATP synthase system is crucial for pneumococcal viability [32] and plays a pivotal role in converting adenosine diphosphate (ADP) and inorganic phosphate (P) into adenosine triphosphate (ATP), an indispensable source of cellular energy. Spn, being a facultative anaerobe, predominantly depends on carbohydrate fermentation to generate energy through glycolysis, for a net gain of two ATP molecules. ATP synthesis is critical for the adaptability of Spn to varying oxygen environments, allowing them to thrive in diverse ecological niches. We observe downregulation of key genes involved in the glycolytic pathway, such as *SPD1823* (−2.2), *SPD0445* (−1.7), *SPD1468* (−1.6), and *SPD1012* (−1.4), which encode enzymes like type I glyceraldehyde-3-phosphate dehydrogenase, phosphoglycerate kinase, phosphoglycerate mutase, and phosphopyruvate hydratase that play a crucial role in converting glucose to pyruvate, a process that yields two molecules of ATP. Collectively, these alterations culminate in a negative impact on the ATP pool within the cellular environment.

#### 3.2.4. AMXT 1501 Treatment Impact on Fatty Acid Synthesis and Polyamine Regulation

**Figure 6:**
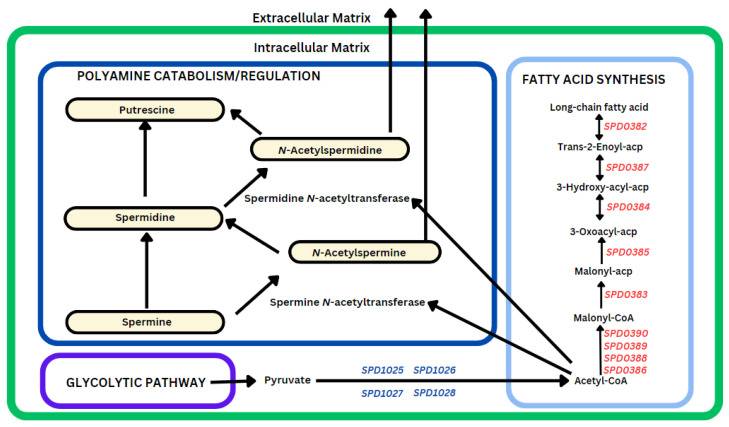
Impact of AMXT 1501 on fatty acid synthesis, polyamine catabolism, and regulation. Genes encoding enzymes that utilize acetyl-CoA, derived from pyruvate, show significant downregulation (shown in red). Specifically, the expression of *SPD0386*, *SPD0388*, *SPD0389*, and *SPD0390*, which encode acetyl-CoA carboxylase biotin carboxyl carrier protein, acetyl-CoA carboxylase biotin carboxylase subunit, acetyl-CoA carboxylase carboxyltransferase subunit beta, and acetyl-CoA carboxylase carboxyl transferase subunit alpha, respectively, involved in the conversion of acetyl-CoA to malonyl-CoA, is hindered. Furthermore, additional critical enzymes in the fatty acid synthesis pathway, including *SPD0383* (ACP *S*-malonyltransferase), *SPD0385* (beta-ketoacyl-ACP synthase II), *SPD0384* (3-oxoacyl-[acyl-carrier-protein] reductase), *SPD0387* (3-hydroxyacyl-ACP dehydratase FabZ), and *SPD0382* (enoyl-[acyl-carrier-protein] reductase FabK), which sequentially catalyze the formation of long-chain fatty acids from malonyl-CoA and its intermediates, are also downregulated. In an alternative pathway responsible for the catabolism and regulation of intracellular polyamines, acetyl-CoA is required to acetylate and remove excess polyamines. Upregulation of *SPD1025* to *SPD1028* (shown in blue) necessary for the conversion of pyruvate to acetyl-CoA supports the notion of cells actively using available acetyl-CoA for polyamine regulation and export from the intracellular matrix rather than fatty acid synthesis.

Genes associated with acetyl-CoA utilization in fatty acid synthesis, including *SPD0382* (−7.1), *SPD0383* (−6.8), *SPD0384* (−5.5), *SPD0385* (−4.3), *SPD0386* (−4.1), *SPD0387* (−4.6), *SPD0388* (−4.3), *SPD0389* (−4.1), and *SPD0390* (−4.0), were downregulated (Figure 6). In contrast, an upregulation is observed in *SPD1025* (2.4), *SPD1026* (1.9), *SPD1027* (2.1), and *SPD1028* (1.7), encoding dihydrolipoyl dehydrogenase, dihydrolipoamide acetyltransferase, alpha-ketoacid dehydrogenase subunit beta, and thiamine pyrophosphate-dependent dehydrogenase E1 component subunit alpha, facilitating the conversion of pyruvate to acetyl-CoA. An alternative pathway for the use of acetyl-CoA involves its activation of spermidine/spermine *N*-acetyltransferase, catalyzing the formation of acetylated spermidine and spermine. This process regulates intracellular polyamine levels by facilitating the export of excess polyamines in their acetylated forms from the cell.

## 4. Discussion

*Streptococcus pneumoniae*, a Gram-positive bacterium, is a significant human pathogen responsible for a spectrum of diseases ranging from mild respiratory infections to severe invasive diseases. While the pneumococcal virulence factors are well documented [33], the role of polyamines in modulating these factors, especially the capsule, has emerged as an interesting area of research. The capsule, a crucial determinant of virulence, plays a pivotal role in immune evasion and the overall survival of the bacterium within the host. Polyamines, being ubiquitous small organic cations, have been implicated in various cellular processes, prompting an exploration of their potential regulatory functions in *S. pneumoniae*. The capsule biosynthesis in *S. pneumoniae* is intricately controlled [34,35], and recent studies indicate that polyamines play a pivotal role in this process [17,18,19]. The positive charge carried by polyamines could interact with the negatively charged capsule polymers, thereby impacting their structure, synthesis, and consequently, virulence. Recent investigations utilizing difluoromethylornithine (DFMO), a well-established polyamine synthesis inhibitor, have revealed regulatory effects on the capsule of pneumococcal D39 [21]. These effects can be attributed to the inhibition of glucose, a key precursor in capsule synthesis. In contrast, the use of AMXT 1501, a polyamine transport inhibitor, is an intriguing alternative for regulating the D39 capsule. Remarkably, this regulation of capsules seems independent of glucose availability [21], presenting a novel avenue for further exploration. This study primarily focuses on gene expression changes that lead to reduced glucose levels in DFMO-treated D39 and increased glucose levels in AMXT 1501-treated D39, despite similar inhibition of capsule production.

The reported gene expression changes within this study unequivocally demonstrate that DFMO effectively inhibits polyamine biosynthesis genes, as illustrated in Figure 1. This inhibition consequently disrupts pathways associated with the import of glucose and the interconversion of other sugars into glucose, as depicted in Figure 2. In stark contrast, AMXT 1501, a compound exclusively targeting polyamine transport while leaving synthesis unaffected, significantly enhances the expression of polyamine and precursor biosynthesis genes, as evident in Figure 3. This augmentation, in turn, results in the increased expression of glucose importers and genes responsible for glucose production, as outlined in Figure 4. These results collectively indicate a direct correlation between cellular glucose levels and the impact of regulation of polyamine biosynthesis. The complex interaction between polyamine metabolism and glucose homeostasis provides valuable insights into the diverse regulatory functions of polyamines in modulating metabolic pathways.

Although we observed a significant downregulation of polyamine and glucose biosynthesis pathways, as well as the inhibition of phosphate and iron import, providing compelling evidence and an explanation for the loss of the capsule and susceptibility of Spn to stress following DFMO treatment, we also noted countermeasures in response to the treatment. These include the upregulation of *SPD1642* (1.4) (ABC transporter permease/substrate-binding protein), associated with osmoprotection and stress response [36], and ribosomal proteins (*SPD0401* (1.7), *SPD0732* (1.5), and *SPD1245* (1.5)) (Supplementary Table S1). However, this adaptive response appears to be insufficient to overcome the deleterious effects of DFMO on pneumococcal capsule production.

Analyzing the alterations in gene expression in cells treated with AMXT 1501, we observed intriguing patterns. Despite the ample availability of glucose, there was notable downregulation of the genes from the glycolytic pathway, responsible for generating ATP, and the ATP synthase system, crucial for converting ADP to ATP, as depicted in Figure 5. ATP synthase functions as a rotary motor, converting proton motive force into rotational energy. The rotational energy produced by ATP synthase is employed to phosphorylate ADP, facilitating the cellular generation of ATP [37,38]. Capsule biosynthesis in pneumococci is an energy-intensive biological process [39,40], suggesting that inhibiting glycolysis and the ATP synthase system would directly impact capsule biosynthesis. The observed upregulation of the two-component regulatory system (CiaRH, *SPD0701*/*SPD0702*), known for negatively regulating competence while positively regulating biofilm formation, antibiotic resistance, thermal adaptation, and tolerance to both acid and oxidative stress [41,42], seems to be influenced by polyamines. This influence may potentially be responsible for the shutdown of energy production and capsule biosynthesis, redirecting resources towards stress responses.

While AMXT 1501 disrupts the ATP synthase system and glycolysis (Figure 5), glucose accumulation is expected and has already been established in our previous work [21]. Increased glucose levels have been reported to enhance polyamine acetylation by reducing the availability of acetyl-CoA for malonyl-CoA synthesis, promoting increased fatty acid oxidation over fatty acid synthesis [43], which is in concordance with our observed inhibition of the fatty acid synthesis pathway (Figure 6). Endothelial cells have been shown to release polyamines that stimulate adipocyte lipolysis, generating free fatty acids that are oxidized for energy to support cell proliferation while improving glucose metabolism [44]. DFMO treatment of neuroblastoma cell lines not only decreased the expression of MYCN, a proto-oncogene, but also resulted in reduced expression of LIN28. LIN28 is reported to suppress let-7 microRNA and is implicated in the regulation of glucose metabolism, promoting aerobic glycolysis, influencing key metabolic enzymes, and impacting stem cell metabolism [45]. In prokaryotic cells, AMXT 1501 was recently shown to have antibacterial activity against Gram-positive and Gram-negative bacteria, including methicillin-resistant *Staphylococcus aureus*, carbapenem-resistant *E. coli*, *Klebsiella pneumoniae*, and *Pseudomonas aeruginosa*. It was reported to reduce biofilm formation and target microbial membrane fatty acid components such as cardiolipin and phosphatidylglycerol [46].

Furthermore, the downregulation of key components involved in iron transport, including *SPD0915* (−1.8) (ABC transporter substrate-binding protein), *SPD0916* (−1.5) (iron ABC transporter permease), *SPD0918* (−1.4) (ABC transporter ATP-binding protein), as well as manganese/zinc transporters *SPD1461* (−4.9) (metal ABC transporter ATP-binding protein), *SPD1462* (−4.0) (metal ABC transporter permease), and *SPD1463* (−5.3) (metal ABC transporter substrate-binding lipoprotein/adhesin PsaA), was observed in AMXT 1501-treated cells (Supplementary Table S2). These components play pivotal roles in facilitating the transport of metals that serve as essential cofactors in redox reactions and stress management processes [30,47,48]. This further strengthens the interplay between capsule biosynthesis and stress responses mediated by polyamines.

In summary, the distinctive mechanisms associated with each polyamine biosynthesis and transport inhibitor highlight the complexity of polyamine-mediated regulation in *S. pneumoniae*, particularly concerning capsule biosynthesis. Notwithstanding this complexity, the discoveries from this study provide valuable insights into potential therapeutic strategies for addressing *S. pneumoniae* infections. Specifically, targeted modulation of virulence factors via polyamine metabolism emerges as a promising avenue for intervention. For translating study findings into practical applications such as drug development, it is critical to understand the specificity and pharmacokinetics of polyamine inhibitors, undesirable off-target effects, and variations in the response among different Spn serotypes.

## 5. Conclusions

In conclusion, this study delves into the intricate regulatory roles of polyamines in Spn, specifically capsule production. Modulation of polyamine metabolism and the impact on eukaryotic pathways are well documented. The intersection of polyamine metabolism and bacterial pathogenesis is an emerging area of investigation, and this study focuses on a human pathogen, *S. pneumoniae*, which poses a significant burden on global health. We report the effect of polyamine synthesis (DFMO) and transport inhibition (AMXT 1501) on pneumococcal gene expression. Our results provide crucial insights into the complex interplay between altered polyamine metabolism, capsule regulation, and stress responses in Spn. This research holds significant implications for the development of targeted small-molecule intervention strategies to modulate virulence factors, offering potential novel alternatives to traditional vaccines and antibiotics. The relevance of our work extends beyond fundamental insights, offering promising prospects for the development of therapeutic strategies with potential clinical applications and ultimately contributing to improved outcomes in the management of Spn-related diseases. Future research directions could explore the translational aspects of these findings, considering clinical applicability, efficacy, and safety for the development of targeted interventions against Spn infections.

## Figures and Tables

**Table 1:** Primers used to conduct qRT-PCR in this study.

Sequence (5′-3′)	Primer Name
CAACTGATGAAGAACTCAAAGAACAC	*gapdh F*
TGGCTCGCTACGATAAGAGATG	*gapdh R*
GGTGTCAGTGTTGGTTTGCG	*SPD0813 F*
TGCACAAGGGTCATAGAGCG	*SPD0813 R*
TGCGGATGATTTCGTCTACAATG	*SPD0811 F*
TCCAGTTCAGGATAGAGTGTTAATAC	*SPD0811 R*
TAAAGACGGTTGGAGTGCCC	*SPD1663 F*
CCCGATTTCCTCACCCATGT	*SPD1663 R*
TTCATCGCAGCCGTAGAACC	*SPD0561 F*
TGGCACAAGCCCAGATTTCA	*SPD0561 R*
CTTGTATAACTCTATTGCCGTCATTG	*SPD0815 F*
GTCATCTGGTATATGGGTCTTTCG	*SPD0815 R*
TTGGAGGTTCACGCTTCGTT	*SPD1496 F*
AATTGGACCCGCCTGAGAAA	*SPD1496 R*
GTTCTCGGAATCTGTTTGGTATCG	*SPD1664 F*
GCTGGGATAACTTGGGCTTGG	*SPD1664 R*

## Data Availability

The datasets generated for this study can be found in the NCBI GEO, accession number: GSE252370.

## Supplementary Materials

The following supporting information can be downloaded at: https://www.mdpi.com/article/10.3390/biom14020178/s1, Table S1: Differentially expressed genes in pneumococcal D39 with DFMO treatment; Table S2: Differentially expressed genes in pneumococcal D39 with AMXT1501 treatment.
