# Supplementary material for: Impact of Difluoromethylornithine and AMXT 1501 on Gene Expression and Capsule Regulation in Streptococcus pneumoniae
Source: Biomolecules. 2024 Feb 2;14(2):178. doi: 10.3390/biom14020178 (PMC10887117; doi:10.3390/biom14020178)
Supplement: Supplementary file 1 [file biomolecules-14-00178-s001.zip › biomolecules-2828800-supplementary.pdf]

Table S1: Differentially expressed genes in pneumococcal D39 with DFMO treatment.

Old_locus_tag	New_locus_tag	Gene	Product	Fold change	FDR p-value
SPD_1180	*SPD_RS06315*		type II CAAX endopeptidase family protein	-29.4	<0.01
SPD_1614	*SPD_RS08605*		phosphate uptake regulator PhoU	-6.7	0.04
SPD_1910	*SPD_RS10125*	*pstS*	substrate-binding domain-containing protein	-5.6	<0.01
SPD_1912	*SPD_RS10135*	*pstA*	phosphate ABC transporter permease PstA	-5.1	<0.01
SPD_1913	*SPD_RS10140*	*pstB*	phosphate ABC transporter ATP-binding protein PstB	-4.9	<0.01
SPD_1911	*SPD_RS10130*	*pstC*	phosphate ABC transporter permease subunit PstC	-4.8	<0.01
SPD_1914	*SPD_RS10145*	*phoU*	phosphate signaling complex protein PhoU	-4.4	<0.01
SPD_1663	*SPD_RS08865*	*treC*	alpha alpha-phosphotrehalase	-3.6	<0.01
SPD_1664	*SPD_RS08875*	*treP*	PTS system trehalose-specific EIIBC component	-3.5	<0.01
SPD_1534	*SPD_RS08165*	*scrB*	sucrose-6-phosphate hydrolase	-3.0	<0.01
	SPD_RS02480		hypothetical protein	-2.9	<0.01
SPD_0473	*SPD_RS02550*	*blpY*	CPBP family intramembrane metalloprotease	-2.8	<0.01
SPD_1535	*SPD_RS08170*	*scrR*	LacI family DNA-binding transcriptional regulator	-2.8	<0.01
SPD_1607	*SPD_RS08575*		iron ABC transporter permease	-2.7	<0.01
SPD_1532	*SPD_RS08150*		sucrose-specific PTS transporter subunit IIBC	-2.5	<0.01
SPD_0691	*SPD_RS03725*		PadR family transcriptional regulator	-2.5	<0.01
SPD_1965	*SPD_RS10405*	*pcpA*	choline-binding protein PcpA	-2.3	<0.01
SPD_1531	*SPD_RS08145*	*scrK*	ROK family protein	-2.1	<0.01
SPD_0138	*SPD_RS00775*		glycosyltransferase family 4 protein	-2.1	0.01
SPD_1834	*SPD_RS09765*	*adhE*	bifunctional acetaldehyde-CoA/alcohol dehydrogenase	-2.1	<0.01
SPD_0069	*SPD_RS00350*		PTS sugar transporter subunit IIA	-2.1	0.05
SPD_1215	*SPD_RS06495*	*amy*	alpha-amylase	-2.0	<0.01
SPD_0459	*SPD_RS02470*	*grpE*	nucleotide exchange factor GrpE	-2.0	<0.01
SPD_1591	*SPD_RS08485*		CsbD family protein	-2.0	0.02
SPD_0460	*SPD_RS02475*	*dnaK*	molecular chaperone DnaK	-1.9	<0.01
SPD_1179	*SPD_RS06305*		protein kinase/lanthionine synthetase C family protein	-1.9	0.01
SPD_0461	*SPD_RS02485*	*dnaJ*	molecular chaperone DnaJ	-1.9	<0.01
SPD_1499	*SPD_RS07965*	*nanB*	neuraminidase NanB	-1.9	0.02
SPD_0458	*SPD_RS02465*	*hrcA*	heat-inducible transcriptional repressor HrcA	-1.8	<0.01
SPD_0338	*SPD_RS01840*		DUF1273 domain-containing protein	-1.8	0.01
SPD_0475	*SPD_RS02565*		type II CAAX endopeptidase family protein	-1.8	0.02
SPD_0068	*SPD_RS00345*		PTS system mannose/fructose/sorbose family transporter subunit IID	-1.8	0.02
SPD_0692	*SPD_RS03730*		hypothetical protein	-1.8	<0.01
SPD_0995	*SPD_RS05340*		YitT family protein	-1.7	0.01
SPD_0827	*SPD_RS04440*	*yabA*	DNA replication initiation control protein YabA	-1.7	0.02
SPD_0661	*SPD_RS03555*	*exp5*	PTS transporter subunit IIBC	-1.6	<0.01
SPD_1577	*SPD_RS08395*		type II toxin-antitoxin system HicB family antitoxin	-1.6	0.03
SPD_0311	*SPD_RS01700*	*dexB*	alpha-glucosidase	-1.6	<0.01
SPD_1121	*SPD_RS05980*		TIGR00341 family protein	-1.5	0.02
SPD_0988	*SPD_RS05310*		YoaK family protein	-1.5	0.04

SPD_0813	*SPD_RS04360*	*nspC*	carboxynorspermidine decarboxylase	-1.5	0.01
SPD_0815	*SPD_RS04370*	*aguB*	N-carbamoylputrescine amidase	-1.5	0.02
SPD_0759	*SPD_RS04070*		GAF domain-containing protein	-1.5	0.01
SPD_1934	*SPD_RS10245*	*malX*	maltodextrin ABC transporter substrate-binding protein	-1.5	0.02
SPD_1227	*SPD_RS06560*	*phoU*	phosphate signaling complex protein PhoU	-1.4	0.04
SPD_1231	*SPD_RS06580*	*pstC*	phosphate ABC transporter permease subunit PstC	-1.3	0.04
SPD_1230	*SPD_RS06575*	*pstA*	phosphate ABC transporter permease PstA	-1.3	0.04
SPD_1644	*SPD_RS08765*		nuclear transport factor 2 family protein	1.4	0.02
SPD_1290	*SPD_RS06890*		amino acid ABC transporter permease	1.4	0.01
SPD_1642	*SPD_RS08755*	*proWX*	ABC transporter permease/substrate-binding protein	1.4	<0.01
SPD_0150	*SPD_RS00840*		amino acid ABC transporter substrate-binding protein	1.4	0.03
SPD_0151	*SPD_RS00845*		MetQ/NlpA family ABC transporter substrate-binding protein	1.4	0.04
SPD_0582	*SPD_RS03150*		DUF3042 family protein	1.5	0.03
SPD_0667	*SPD_RS03590*	*sodA*	superoxide dismutase SodA	1.5	0.02
SPD_0732	*SPD_RS03945*	*rpsT*	30S ribosomal protein S20	1.5	0.03
SPD_1943	*SPD_RS10295*		hypothetical protein	1.5	0.02
SPD_1245	*SPD_RS06650*	*rpsU*	30S ribosomal protein S21	1.5	0.04
SPD_0687	*SPD_RS03705*		ABC transporter ATP-binding protein	1.5	0.01
SPD_0039	*SPD_RS00185*		CoA-binding protein	1.5	<0.01
SPD_0987	*SPD_RS05305*		YbaB/EbfC family nucleoid-associated protein	1.6	0.01
SPD_0541	*SPD_RS02900*		nitroreductase family protein	1.6	<0.01
SPD_1567	*SPD_RS08335*	*trxA*	thioredoxin	1.6	0.02
SPD_2033	*SPD_RS10755*	*raiA*	ribosome-associated translation inhibitor RaiA	1.6	0.02
SPD_0266	*SPD_RS01450*		Cof-type HAD-IIB family hydrolase	1.6	0.01
SPD_1528	*SPD_RS08125*		ABC transporter ATP-binding protein	1.6	0.03
SPD_1295	*SPD_RS06915*		hemolysin III family protein	1.6	0.01
SPD_0261	*SPD_RS01425*	*pepC*	aminopeptidase C	1.6	<0.01
SPD_0523	*SPD_RS02805*	*vex3*	ABC transporter permease subunit Vex3	1.6	<0.01
SPD_0381	*SPD_RS02060*	*acpP*	acyl carrier protein	1.6	0.01
SPD_0896	*SPD_RS04820*	*mscL*	large conductance mechanosensitive channel protein MscL	1.7	<0.01
SPD_1415	*SPD_RS07525*		FAD-containing oxidoreductase	1.7	0.01
SPD_1010	*SPD_RS05420*		YueI family protein	1.7	0.03
SPD_0401	*SPD_RS02170*	*rpmB*	50S ribosomal protein L28	1.7	<0.01
SPD_1706	*SPD_RS09085*		DUF402 domain-containing protein	1.7	<0.01
SPD_1793	*SPD_RS09550*		universal stress protein	1.7	0.02
SPD_0521	*SPD_RS02795*	*vex1*	ABC transporter permease	1.8	<0.01
SPD_1527	*SPD_RS08120*		ABC transporter permease	1.8	<0.01
SPD_0997	*SPD_RS05350*	*hup*	HU family DNA-binding protein	1.8	<0.01
SPD_0522	*SPD_RS02800*	*vex2*	ABC transporter ATP-binding subunit Vex2	1.8	<0.01
	SPD_RS06310		hypothetical protein	1.8	<0.01
SPD_1566	*SPD_RS08330*		DUF4649 family protein	1.8	<0.01

SPD_1294	*SPD_RS06910*		DUF1836 domain-containing protein	1.8	0.03
SPD_2069	*SPD_RS10950*		ParB/RepB/Spo0J family partition protein	1.9	<0.01
SPD_0684	*SPD_RS03690*		biotin transporter BioY	1.9	0.01
SPD_2013	*SPD_RS10650*	*glpK*	glycerol kinase GlpK	1.9	0.02
SPD_1327	*SPD_RS07065*	*bta*	thioredoxin	2.0	<0.01
SPD_1360	*SPD_RS07235*		CsbD family protein	2.0	<0.01
SPD_2068	*SPD_RS10945*		trypsin-like peptidase domain-containing protein	2.0	<0.01
SPD_1649	*SPD_RS08795*		ABC transporter permease	2.3	<0.01
SPD_1651	*SPD_RS08805*		ATP-binding cassette domain-containing protein	2.4	<0.01
SPD_1526	*SPD_RS08115*		membrane protein	2.4	<0.01
SPD_1652	*SPD_RS08810*		siderophore ABC transporter substrate-binding protein	2.4	<0.01
SPD_1650	*SPD_RS08800*		iron chelate uptake ABC transporter family permease subunit	2.4	<0.01
SPD_1525	*SPD_RS08110*		ABC transporter ATP-binding protein	2.4	<0.01
SPD_1041	*SPD_RS05590*	*nrdH*	glutaredoxin-like protein NrdH	2.5	<0.01
SPD_1524	*SPD_RS08105*		GntR family transcriptional regulator	2.6	<0.01

Table S2: Differentially expressed genes in pneumococcal D39 with AMXT1501 treatment.

**Old_locus_tag**	**New_locus_tag**	**Gene**	**Product**	**Fold change**	**FDR p-value**
SPD_1699	*SPD_RS09050*	*rrfB*	5S ribosomal RNA	-27.5	<0.01
SPD_1813	*SPD_RS09650*	*rrfC*	5S ribosomal RNA	-26.9	<0.01
SPD_1890	*SPD_RS10025*	*rrfD*	5S ribosomal RNA	-26.2	<0.01
SPD_1180	*SPD_RS06315*		type II CAAX endopeptidase family protein	-25.0	<0.01
SPD_0019	*SPD_RS00090*	*rrfA*	5S ribosomal RNA	-17.6	<0.01
SPD_1682	*SPD_RS08965*		tRNA-Ser	-9.0	<0.01
SPD_1727	*SPD_RS09190*		hypothetical protein	-8.9	<0.01
SPD_1726	*SPD_RS09185*	*ply*	cholesterol-dependent cytolysin pneumolysin	-8.5	<0.01
SPD_0264	*SPD_RS01440*	*manL*	PTS sugar transporter subunit IIB	-8.2	<0.01
SPD_0381	*SPD_RS02060*	*acpP*	acyl carrier protein	-8.1	<0.01
SPD_0382	*SPD_RS02065*	*fabK*	enoyl-[acyl-carrier-protein] reductase FabK	-7.1	<0.01
SPD_1729	*SPD_RS09200*		DUF4231 domain-containing protein	-6.9	<0.01
SPD_0383	*SPD_RS02070*	*fabD*	ACP S-malonyltransferase	-6.8	<0.01
SPD_1728	*SPD_RS09195*		hypothetical protein	-6.7	<0.01
SPD_1695	*SPD_RS09030*		tRNA-Leu	-6.2	0.01
SPD_0401	*SPD_RS02170*	*rpmB*	50S ribosomal protein L28	-6.0	<0.01
SPD_0262	*SPD_RS01430*		PTS system mannose/fructose/sorbose family transporter subunit IID	-5.7	<0.01
SPD_0263	*SPD_RS01435*	*manM*	PTS mannose/fructose/sorbose transporter subunit IIC	-5.6	<0.01
SPD_0997	*SPD_RS05350*	*hup*	HU family DNA-binding protein	-5.6	<0.01

SPD_0384	*SPD_RS02075*	*fabG*	3-oxoacyl-[acyl-carrier-protein] reductase	-5.5	<0.01
SPD_1463	*SPD_RS07780*		metal ABC transporter substrate-binding lipoprotein/adhesin PsaA	-5.3	<0.01
SPD_0391	*SPD_RS02115*		biofilm-regulating peptide BriC	-5.0	<0.01
SPD_1245	*SPD_RS06650*	*rpsU*	30S ribosomal protein S21	-4.9	<0.01
SPD_1461	*SPD_RS07770*	*psaB*	metal ABC transporter ATP-binding protein	-4.9	<0.01
SPD_0378	*SPD_RS02045*		enoyl-CoA hydratase	-4.8	<0.01
SPD_0387	*SPD_RS02090*	*fabZ*	3-hydroxyacyl-ACP dehydratase FabZ	-4.6	<0.01
SPD_0385	*SPD_RS02080*	*fabF*	beta-ketoacyl-ACP synthase II	-4.3	<0.01
SPD_0388	*SPD_RS02095*	*accC*	acetyl-CoA carboxylase biotin carboxylase subunit	-4.3	<0.01
SPD_0188	*SPD_RS11265*		hypothetical protein	-4.2	<0.01
SPD_0386	*SPD_RS02085*	*accB*	acetyl-CoA carboxylase biotin carboxyl carrier protein	-4.1	<0.01
SPD_0389	*SPD_RS02100*	*accD*	acetyl-CoA carboxylase carboxyltransferase subunit beta	-4.1	<0.01
SPD_1462	*SPD_RS07775*	*psaC*	metal ABC transporter permease	-4.0	<0.01
SPD_0390	*SPD_RS02105*	*accA*	acetyl-CoA carboxylase carboxyl transferase subunit alpha	-4.0	<0.01
SPD_1692	*SPD_RS09015*		tRNA-Leu	-3.9	0.01
SPD_1834	*SPD_RS09765*		bifunctional acetaldehyde-CoA/alcohol dehydrogenase	-3.8	<0.01
SPD_1806	*SPD_RS09615*		tRNA-Leu	-3.8	0.01
SPD_1964	*SPD_RS10400*	*rpmG*	50S ribosomal protein L33	-3.8	<0.01
SPD_0732	*SPD_RS03945*	*rpsT*	30S ribosomal protein S20	-3.8	<0.01
SPD_1790	*SPD_RS09535*	*rpmH*	50S ribosomal protein L34	-3.7	<0.01
SPD_0191	*SPD_RS01060*		phosphoribulokinase	-3.7	<0.01
SPD_1963	*SPD_RS10395*	*rpmF*	50S ribosomal protein L32	-3.6	<0.01
SPD_0558	*SPD_RS03010*	*prtA*	S8 family serine peptidase	-3.5	<0.01
SPD_0410	*SPD_RS02220*		hypothetical protein	-3.5	<0.01
SPD_0189	*SPD_RS01050*		GNAT family N-acetyltransferase	-3.3	<0.01
SPD_1607	*SPD_RS08575*		iron ABC transporter permease	-3.3	<0.01
	SPD_RS02125		CPBP family intramembrane metalloprotease	-3.2	<0.01
SPD_0771	*SPD_RS04130*	*lacR1*	DeoR/GlpR family DNA-binding transcription regulator	-3.0	<0.01
	SPD_RS02410		hypothetical protein	-3.0	0.01
SPD_0190	*SPD_RS01055*	*nrdG*	anaerobic ribonucleoside-triphosphate reductase activating protein	-3.0	<0.01
SPD_1154	*SPD_RS06155*	*rpmE*	type B 50S ribosomal protein L31	-3.0	<0.01
SPD_0674	*SPD_RS03635*	*rpsP*	30S ribosomal protein S16	-3.0	<0.01
SPD_0898	*SPD_RS04830*		QueT transporter family protein	-2.9	<0.01
SPD_0772	*SPD_RS04135*		1-phosphofructokinase	-2.9	<0.01
SPD_1899	*SPD_RS10070*		gamma-glutamyl-gamma-aminobutyrate hydrolase family protein	-2.9	<0.01
SPD_1662	*SPD_RS08860*		YneF family protein	-2.9	<0.01
SPD_1674	*SPD_RS11760*		hypothetical protein	-2.8	<0.01
SPD_0773	*SPD_RS04140*		fructose-specific PTS transporter subunit EIIC	-2.8	<0.01

	SPD_RS06310		hypothetical protein	-2.7	<0.01
SPD_0861	*SPD_RS04635*	*secG*	preprotein translocase subunit SecG	-2.7	<0.01
SPD_1235	*SPD_RS06600*		UPF0223 family protein	-2.7	<0.01
SPD_0896	*SPD_RS04820*	*mscL*	large conductance mechanosensitive channel protein MscL	-2.6	<0.01
SPD_1606	*SPD_RS08570*		MgtC/SapB family protein	-2.4	<0.01
SPD_1294	*SPD_RS06910*		DUF1836 domain-containing protein	-2.4	<0.01
SPD_1600	*SPD_RS08540*	*trpD*	anthranilate phosphoribosyltransferase	-2.4	<0.01
SPD_1644	*SPD_RS08765*		nuclear transport factor 2 family protein	-2.3	<0.01
SPD_1725	*SPD_RS09175*		YebC/PmpR family DNA-binding transcriptional regulator	-2.3	<0.01
SPD_1645	*SPD_RS08770*		MarR family transcriptional regulator	-2.3	<0.01
SPD_0373	*SPD_RS02015*		carboxymuconolactone decarboxylase family protein	-2.3	<0.01
SPD_0187	*SPD_RS01040*	*nrdD*	anaerobic ribonucleoside-triphosphate reductase	-2.3	<0.01
SPD_0492	*SPD_RS02650*		GNAT family protein	-2.3	<0.01
SPD_0649	*SPD_RS03490*	*upp*	uracil phosphoribosyltransferase	-2.3	<0.01
SPD_0380	*SPD_RS02055*	*fabH*	ketoacyl-ACP synthase III	-2.2	<0.01
SPD_1823	*SPD_RS09705*	*gap*	type I glyceraldehyde-3-phosphate dehydrogenase	-2.2	<0.01
SPD_1295	*SPD_RS06915*		hemolysin III family protein	-2.2	<0.01
SPD_1943	*SPD_RS10295*		hypothetical protein	-2.2	<0.01
SPD_0848	*SPD_RS04565*	*rpmI*	50S ribosomal protein L35	-2.2	<0.01
SPD_1040	*SPD_RS05585*	*ptsH*	phosphocarrier protein HPr	-2.1	<0.01
SPD_1965	*SPD_RS10405*	*pcpA*	choline-binding protein PcpA	-2.1	<0.01
SPD_0379	*SPD_RS02050*		MarR family transcriptional regulator	-2.1	<0.01
	SPD_RS10390		PTS ascorbate transporter subunit IIC	-2.1	<0.01
SPD_1444	*SPD_RS07680*	*thrS*	threonine--tRNA ligase	-2.1	<0.01
SPD_0491	*SPD_RS02645*		flavin reductase	-2.1	<0.01
SPD_0849	*SPD_RS04570*	*rplT*	50S ribosomal protein L20	-2.1	<0.01
SPD_1598	*SPD_RS08530*	*trpF*	phosphoribosylanthranilate isomerase	-2.1	<0.01
SPD_0398	*SPD_RS02155*	*gatC*	Asp-tRNA(Asn)/Glu-tRNA(Gln) amidotransferase subunit GatC	-2.0	<0.01
SPD_0274	*SPD_RS01490*	*rplM*	50S ribosomal protein L13	-2.0	<0.01
SPD_1187	*SPD_RS06345*	*rplL*	50S ribosomal protein L7/L12	-2.0	<0.01
SPD_1931	*SPD_RS10225*		membrane protein	-2.0	<0.01
SPD_1596	*SPD_RS08520*	*trpA*	tryptophan synthase subunit alpha	-2.0	<0.01
SPD_0275	*SPD_RS01495*	*rpsI*	30S ribosomal protein S9	-2.0	<0.01
SPD_2043	*SPD_RS10810*		CHAP domain-containing protein	-2.0	<0.01
SPD_1601	*SPD_RS08545*	*trpG*	aminodeoxychorismate/anthranilate synthase component II	-2.0	<0.01
SPD_1673	*SPD_RS08925*	*gtfA*	sucrose phosphorylase	-2.0	<0.01
SPD_1439	*SPD_RS07645*	*rpsO*	30S ribosomal protein S15	-2.0	<0.01
SPD_1599	*SPD_RS08535*	*trpC*	indole-3-glycerol phosphate synthase TrpC	-2.0	<0.01
SPD_0083	*SPD_RS00445*	*rpsD*	30S ribosomal protein S4	-2.0	<0.01
SPD_1443	*SPD_RS07675*		MazG-like protein	-2.0	0.02

	SPD_RS01185		50S ribosomal protein L36	-2.0	<0.01
SPD_1675	*SPD_RS08930*	*rafG*	carbohydrate ABC transporter permease	-2.0	<0.01
SPD_1360	*SPD_RS07235*		CsbD family protein	-2.0	<0.01
SPD_0847	*SPD_RS04560*	*infC*	translation initiation factor IF-3	-2.0	<0.01
SPD_1643	*SPD_RS08760*	*proV*	ABC transporter ATP-binding protein	-2.0	<0.01
SPD_1524	*SPD_RS08105*		GntR family transcriptional regulator	-1.9	<0.01
SPD_1608	*SPD_RS08580*		ABC transporter ATP-binding protein	-1.9	0.01
	SPD_RS04950		restriction endonuclease subunit S	-1.9	0.04
SPD_0718	*SPD_RS03865*		YkuJ family protein	-1.9	<0.01
SPD_1597	*SPD_RS08525*	*trpB*	tryptophan synthase subunit beta	-1.9	<0.01
SPD_1402	*SPD_RS07455*		DNA starvation/stationary phase protection protein	-1.9	<0.01
SPD_0526	*SPD_RS02820*	*fba*	fructose-bisphosphate aldolase	-1.9	<0.01
SPD_1409	*SPD_RS07490*		sn-glycerol-3-phosphate ABC transporter ATP-binding protein UgpC	-1.9	<0.01
SPD_0675	*SPD_RS03640*		RNA-binding protein KphA	-1.9	<0.01
SPD_1632	*SPD_RS08705*		PaaI family thioesterase	-1.9	<0.01
SPD_1427	*SPD_RS07585*	*phnA*	zinc ribbon domain-containing protein YjdM	-1.9	<0.01
SPD_0636	*SPD_RS03430*	*spxB*	pyruvate oxidase	-1.9	<0.01
SPD_1851	*SPD_RS09845*	*rnpA*	ribonuclease P protein component	-1.9	<0.01
SPD_0004	*SPD_RS00020*		redox-regulated ATPase YchF	-1.9	<0.01
SPD_0094	*SPD_RS00505*		DUF4097 domain-containing protein	-1.8	<0.01
SPD_0915	*SPD_RS04930*		ABC transporter substrate-binding protein	-1.8	<0.01
SPD_1895	*SPD_RS10050*		cytidine deaminase	-1.8	<0.01
SPD_0365	*SPD_RS01975*	*tig*	trigger factor	-1.8	<0.01
SPD_0219	*SPD_RS01205*	*rplQ*	50S ribosomal protein L17	-1.8	<0.01
SPD_0039	*SPD_RS00185*		CoA-binding protein	-1.8	<0.01
SPD_0093	*SPD_RS00500*		membrane protein	-1.8	<0.01
SPD_1472	*SPD_RS07825*	*ileS*	isoleucine--tRNA ligase	-1.8	<0.01
SPD_0091	*SPD_RS00490*		rhodanese-related sulfurtransferase	-1.8	<0.01
SPD_0334	*SPD_RS01815*	*aliA*	peptide ABC transporter substrate-binding protein	-1.8	<0.01
SPD_1339	*SPD_RS07130*	*atpF*	F0F1 ATP synthase subunit B	-1.8	<0.01
SPD_1233	*SPD_RS06590*		RsmF rRNA methyltransferase first C-terminal domain-containing protein	-1.8	<0.01
SPD_1874	*SPD_RS09945*		LysM domain-containing protein	-1.8	0.01
SPD_1426	*SPD_RS07580*		Pr6Pr family membrane protein	-1.8	<0.01
SPD_0216	*SPD_RS01190*	*rpsM*	30S ribosomal protein S13	-1.8	<0.01
SPD_0150	*SPD_RS00840*		amino acid ABC transporter substrate-binding protein	-1.8	<0.01
SPD_1368	*SPD_RS07275*	*rpsR*	30S ribosomal protein S18	-1.8	<0.01
SPD_2042	*SPD_RS10805*	*rpsB*	30S ribosomal protein S2	-1.8	<0.01
SPD_1928	*SPD_RS10210*		DUF6110 family protein	-1.8	<0.01
SPD_0104	*SPD_RS00555*		LysM domain-containing protein	-1.8	0.02
SPD_0155	*SPD_RS00865*		MptD family putative ECF transporter S component	-1.7	<0.01

SPD_1236	*SPD_RS06605*	*spx*	Spx/MgsR family RNA polymerase-binding regulatory protein	-1.7	<0.01
SPD_1792	*SPD_RS09545*		hypothetical protein	-1.7	0.02
SPD_2056	*SPD_RS10875*	*trpS*	tryptophan--tRNA ligase	-1.7	<0.01
SPD_0340	*SPD_RS01850*	*rnpB*	RNase P RNA component class B	-1.7	<0.01
SPD_0445	*SPD_RS02390*	*pgk*	phosphoglycerate kinase	-1.7	<0.01
	SPD_RS09690		50S ribosomal protein L33	-1.7	0.01
SPD_0991	*SPD_RS05325*	*rpmA*	50S ribosomal protein L27	-1.7	<0.01
SPD_0097	*SPD_RS00520*		CynX/NimT family MFS transporter	-1.7	<0.01
SPD_0954	*SPD_RS05140*		tryptophan ABC transporter substrate-binding protein	-1.7	<0.01
SPD_1602	*SPD_RS08550*	*trpE*	anthranilate synthase component I	-1.7	0.01
SPD_0267	*SPD_RS01455*		NCS2 family permease	-1.7	<0.01
SPD_1642	*SPD_RS08755*	*proWX*	ABC transporter permease/substrate-binding protein	-1.7	<0.01
SPD_0317	*SPD_RS01725*	*cps2C*	capsular polysaccharide biosynthesis protein	-1.7	<0.01
SPD_1430	*SPD_RS07600*	*fer*	ferredoxin	-1.7	0.01
SPD_0195	*SPD_RS01080*	*rplW*	50S ribosomal protein L23	-1.7	<0.01
SPD_1188	*SPD_RS06350*	*rplJ*	50S ribosomal protein L10	-1.7	<0.01
SPD_1303	*SPD_RS06950*		DUF896 family protein	-1.7	<0.01
SPD_1369	*SPD_RS07280*	*ssb*	single-stranded DNA-binding protein SsbA	-1.6	<0.01
SPD_0144	*SPD_RS00810*		XRE/MutR family transcriptional regulator	-1.6	<0.01
SPD_1350	*SPD_RS07185*		hypothetical protein	-1.6	<0.01
SPD_1634	*SPD_RS08715*	*galK*	galactokinase	-1.6	<0.01
SPD_1407	*SPD_RS07480*	*apt*	adenine phosphoribosyltransferase	-1.6	<0.01
SPD_1864	*SPD_RS09900*		DUF1033 family protein	-1.6	0.02
SPD_1587	*SPD_RS08460*		virulence factor transcriptional regulator MgaSpn	-1.6	0.04
SPD_0739	*SPD_RS03980*		BMP family protein	-1.6	<0.01
SPD_1468	*SPD_RS07805*		phosphoglycerate mutase	-1.6	<0.01
SPD_1334	*SPD_RS07105*	*atpC*	F0F1 ATP synthase subunit epsilon	-1.6	<0.01
SPD_0184	*SPD_RS01020*		SP_0198 family lipoprotein	-1.6	<0.01
SPD_1338	*SPD_RS07125*	*atpH*	F0F1 ATP synthase subunit delta	-1.6	<0.01
SPD_0582	*SPD_RS03150*		DUF3042 family protein	-1.6	<0.01
SPD_1293	*SPD_RS06905*		GNAT family N-acetyltransferase	-1.6	<0.01
SPD_0271	*SPD_RS01475*	*folE*	GTP cyclohydrolase I FolE	-1.6	<0.01
SPD_0761	*SPD_RS04080*		DUF3272 domain-containing protein	-1.6	0.01
SPD_0095	*SPD_RS00510*		DUF1700 domain-containing protein	-1.6	0.02
SPD_1010	*SPD_RS05420*		YueI family protein	-1.6	0.02
SPD_0494	*SPD_RS02660*	*valS*	valine--tRNA ligase	-1.6	<0.01
SPD_0541	*SPD_RS02900*		nitroreductase family protein	-1.6	<0.01
SPD_1672	*SPD_RS08920*		O-antigen ligase	-1.6	0.02
SPD_1633	*SPD_RS08710*	*galT-2*	UDP-glucose--hexose-1-phosphate uridylyltransferase	-1.6	<0.01
SPD_0987	*SPD_RS05305*		YbaB/EbfC family nucleoid-associated protein	-1.6	<0.01

SPD_1429	*SPD_RS07595*		hypothetical protein	-1.6	<0.01
SPD_0703	*SPD_RS03785*		DUF3270 domain-containing protein	-1.6	0.01
SPD_0436	*SPD_RS02345*		'tRNA (cytidine(34)-2''-O)-methyltransferase'	-1.6	<0.01
SPD_1706	*SPD_RS09085*		DUF402 domain-containing protein	-1.6	<0.01
SPD_0064	*SPD_RS00325*		GntR family transcriptional regulator	-1.6	<0.01
SPD_1305	*SPD_RS06960*	*glyQ*	glycine--tRNA ligase subunit alpha	-1.6	<0.01
SPD_0192	*SPD_RS01065*	*rpsJ*	30S ribosomal protein S10	-1.6	0.01
SPD_0504	*SPD_RS02710*	*pheS*	phenylalanine--tRNA ligase subunit alpha	-1.6	<0.01
SPD_0990	*SPD_RS05320*		ribosomal-processing cysteine protease Prp	-1.6	<0.01
SPD_0266	*SPD_RS01450*		Cof-type HAD-IIB family hydrolase	-1.6	<0.01
SPD_0218	*SPD_RS01200*	*rpoA*	DNA-directed RNA polymerase subunit alpha	-1.6	<0.01
SPD_1905	*SPD_RS10100*	*argS*	arginine--tRNA ligase	-1.6	0.03
SPD_1630	*SPD_RS08695*		DpnD/PcfM family protein	-1.5	0.04
SPD_1304	*SPD_RS06955*	*glyS*	glycine--tRNA ligase subunit beta	-1.5	<0.01
SPD_0490	*SPD_RS02640*		helicase	-1.5	<0.01
SPD_0878	*SPD_RS04730*		chromosome segregation protein RocS	-1.5	<0.01
SPD_1959	*SPD_RS10370*		PTS ascorbate transporter subunit IIC	-1.5	0.02
SPD_1949	*SPD_RS11800*		hypothetical protein	-1.5	0.04
SPD_0956	*SPD_RS05150*		ABC transporter ATP-binding protein	-1.5	0.05
SPD_1345	*SPD_RS07160*	*greA*	transcription elongation factor GreA	-1.5	<0.01
SPD_1336	*SPD_RS07115*	*atpG*	F0F1 ATP synthase subunit gamma	-1.5	<0.01
SPD_0151	*SPD_RS00845*		MetQ/NlpA family ABC transporter substrate-binding protein	-1.5	<0.01
SPD_0550	*SPD_RS02945*	*rplK*	50S ribosomal protein L11	-1.5	0.02
SPD_1525	*SPD_RS08110*		ABC transporter ATP-binding protein	-1.5	0.03
SPD_1148	*SPD_RS06125*	*rplS*	50S ribosomal protein L19	-1.5	<0.01
SPD_0916	*SPD_RS04935*		iron ABC transporter permease	-1.5	0.01
SPD_0338	*SPD_RS01840*		DUF1273 domain-containing protein	-1.5	0.05
SPD_0265	*SPD_RS01445*		alcohol dehydrogenase AdhP	-1.5	<0.01
SPD_2041	*SPD_RS10800*	*tsf*	translation elongation factor Ts	-1.5	<0.01
SPD_1907	*SPD_RS10110*		cupin domain-containing protein	-1.5	<0.01
SPD_0217	*SPD_RS01195*	*rpsK*	30S ribosomal protein S11	-1.5	<0.01
SPD_1039	*SPD_RS05580*	*ptsI*	phosphoenolpyruvate--protein phosphotransfer-ase	-1.5	0.01
SPD_0668	*SPD_RS03595*		YutD family protein	-1.5	0.02
	SPD_RS08340		preQ(1) synthase	-1.5	0.04
SPD_2052	*SPD_RS10855*		pitrilysin family protein	-1.5	<0.01
SPD_0040	*SPD_RS00190*		YeiH family protein	-1.5	0.01
SPD_0193	*SPD_RS01070*	*rplC*	50S ribosomal protein L3	-1.5	0.03
SPD_0754	*SPD_RS04045*		DUF2969 domain-containing protein	-1.5	0.05
SPD_0201	*SPD_RS01110*	*rpmC*	50S ribosomal protein L29	-1.5	0.02
SPD_0395	*SPD_RS02140*	*efp*	elongation factor P	-1.5	0.02
SPD_0215	*SPD_RS01180*	*infA*	translation initiation factor IF-1	-1.5	0.03

SPD_0335	*SPD_RS01825*		SpGH101 family endo-alpha-N-acetylgalactosaminidase	-1.4	0.01
SPD_0375	*SPD_RS02025*	*serS*	serine--tRNA ligase	-1.4	0.01
SPD_1896	*SPD_RS10055*	*gltX*	glutamate--tRNA ligase	-1.4	<0.01
SPD_1425	*SPD_RS07575*		MFS transporter	-1.4	0.01
SPD_0686	*SPD_RS03700*		efflux RND transporter periplasmic adaptor subunit	-1.4	0.01
SPD_1018	*SPD_RS05470*	*iga*	immunoglobulin A1 protease	-1.4	<0.01
SPD_1819	*SPD_RS09680*	*nusG*	transcription termination/antitermination protein NusG	-1.4	0.01
SPD_1012	*SPD_RS05425*	*eno*	phosphopyruvate hydratase	-1.4	0.03
SPD_1292	*SPD_RS06900*	*ogt*	methylated-DNA--[protein]-cysteine S-methyltransferase	-1.4	0.03
SPD_0551	*SPD_RS02950*	*rplA*	50S ribosomal protein L1	-1.4	0.01
SPD_0197	*SPD_RS01090*	*rpsS*	30S ribosomal protein S19	-1.4	0.02
SPD_1340	*SPD_RS07135*	*atpB*	F0F1 ATP synthase subunit A	-1.4	0.02
SPD_1569	*SPD_RS08355*		aquaporin	-1.4	0.05
SPD_1776	*SPD_RS09455*	*purR*	pur operon repressor	-1.4	<0.01
SPD_1337	*SPD_RS07120*	*atpA*	F0F1 ATP synthase subunit alpha	-1.4	<0.01
SPD_1869	*SPD_RS09925*		DUF975 family protein	-1.4	0.04
SPD_1372	*SPD_RS10980*		VOC family protein	-1.4	0.03
SPD_0207	*SPD_RS01140*	*rpsH*	30S ribosomal protein S8	-1.4	0.04
SPD_1460	*SPD_RS07765*	*pepO*	endopeptidase PepO	-1.4	0.01
SPD_0918	*SPD_RS04945*		ABC transporter ATP-binding protein	-1.4	0.02
SPD_1374	*SPD_RS07300*		DUF5590 domain-containing protein	-1.4	0.02
SPD_1397	*SPD_RS07430*		RidA family protein	-1.4	0.02
SPD_0740	*SPD_RS03985*		ABC transporter ATP-binding protein	-1.4	0.01
SPD_0238	*SPD_RS01295*	*leuS*	leucine--tRNA ligase	-1.4	0.01
SPD_0315	*SPD_RS01715*	*cps2A*	capsular polysaccharide biosynthesis protein Cps4A	-1.4	0.02
SPD_0593	*SPD_RS03205*		translational GTPase TypA	-1.4	0.03
SPD_0251	*SPD_RS01370*	*rpsL*	30S ribosomal protein S12	-1.4	0.03
SPD_1274	*SPD_RS06810*	*guaA*	glutamine-hydrolyzing GMP synthase	-1.4	0.01
SPD_1762	*SPD_RS09380*		DNA-entry nuclease EndA	-1.4	0.01
SPD_2017	*SPD_RS10670*	*cbpA*	choline-binding protein CbpA	-1.4	0.02
SPD_0126	*SPD_RS00695*	*pspA*	pneumococcal surface protein A	-1.4	0.02
SPD_1849	*SPD_RS09835*		RNA-binding cell elongation regulator EloR	-1.4	0.02
SPD_0700	*SPD_RS03770*	*pepN*	M1 family metallopeptidase	-1.4	0.04
SPD_1431	*SPD_RS07605*		glycosyltransferase family 2 protein	-1.3	0.01
SPD_1201	*SPD_RS06420*	*licD3*	phosphorylcholine transferase LicD	-1.3	0.03
SPD_0429	*SPD_RS02315*		TrkH family potassium uptake protein	-1.3	0.04
SPD_0394	*SPD_RS02135*		Asp23/Gls24 family envelope stress response protein	-1.3	0.04
SPD_0393	*SPD_RS02130*	*nusB*	transcription antitermination factor NusB	-1.3	0.01
SPD_0688	*SPD_RS03710*		ABC transporter permease	-1.3	0.03

SPD_0548	*SPD_RS02935*		HIT family protein	-1.3	0.03
SPD_0441	*SPD_RS02370*		DNA-directed RNA polymerase subunit delta	-1.3	0.02
SPD_0206	*SPD_RS01135*	*rpsN*	30S ribosomal protein S14	-1.3	0.04
SPD_0547	*SPD_RS02930*		M42 family metallopeptidase	-1.3	0.04
SPD_1768	*SPD_RS09415*	*asnA*	aspartate--ammonia ligase	-1.3	0.02
SPD_1068	*SPD_RS05720*	*udk*	uridine kinase	-1.3	0.04
SPD_1079	*SPD_RS05775*		DEAD/DEAH box helicase family protein	1.3	0.02
SPD_1358	*SPD_RS07225*		polysaccharide biosynthesis protein	1.3	0.03
SPD_1383	*SPD_RS07355*		cation-translocating P-type ATPase	1.3	0.04
SPD_1922	*SPD_RS10180*		N-acetyldiaminopimelate deacetylase	1.3	0.04
SPD_0864	*SPD_RS04650*	*tehB*	SAM-dependent methyltransferase TehB	1.3	0.03
SPD_1704	*SPD_RS09075*	*rumA-2*	23S rRNA (uracil(1939)-C(5))-methyltransferase RlmD	1.3	0.04
SPD_1209	*SPD_RS06460*	*aroB*	3-dehydroquinate synthase	1.3	0.05
SPD_0525	*SPD_RS02815*	*vncS*	sensor histidine kinase VncS	1.4	0.02
SPD_0259	*SPD_RS01410*		GlsB/YeaQ/YmgE family stress response membrane protein	1.4	0.04
SPD_1203	*SPD_RS06430*		prephenate dehydratase	1.4	0.01
SPD_2022	*SPD_RS10700*		ATP-dependent Clp protease ATP-binding subunit	1.4	0.01
SPD_0241	*SPD_RS01315*	*ruvB*	Holliday junction branch migration DNA helicase RuvB	1.4	0.01
SPD_0539	*SPD_RS02890*		metallophosphoesterase family protein	1.4	0.01
SPD_0468	*SPD_RS02520*	*blpR*	response regulator transcription factor	1.4	0.03
SPD_0815	*SPD_RS04370*		N-carbamoylputrescine amidase	1.4	0.03
SPD_1204	*SPD_RS06435*	*aroK*	shikimate kinase	1.4	0.04
SPD_1287	*SPD_RS06875*	*trxB*	thioredoxin-disulfide reductase	1.4	0.01
SPD_2037	*SPD_RS10775*	*cysK*	cysteine synthase A	1.4	0.02
SPD_0082	*SPD_RS00440*		HAMP domain-containing sensor histidine kinase	1.4	0.03
SPD_0960	*SPD_RS05170*	*cpoA*	alpha-galactosylglucosyldiacylglycerol synthase	1.4	<0.01
SPD_1124	*SPD_RS06000*	*licB*	DMT family transporter	1.4	0.04
SPD_1902	*SPD_RS10085*		multidrug efflux ABC transporter subunit PatA	1.4	0.01
SPD_0535	*SPD_RS02870*	*murM*	peptidoglycan bridge formation alanyltransferase MurM	1.4	0.01
SPD_0661	*SPD_RS03555*	*exp5*	PTS transporter subunit IIBC	1.4	0.01
SPD_1084	*SPD_RS05800*		cell wall metabolism sensor histidine kinase VicK	1.4	<0.01
SPD_1192	*SPD_RS06370*		ABC transporter ATP-binding protein	1.4	0.02
SPD_0886	*SPD_RS04765*		thiol-disulfide oxidoreductase-associated lipoprotein SdbB	1.4	0.01
SPD_0642	*SPD_RS03455*		sodium-dependent transporter	1.4	0.04
SPD_0311	*SPD_RS01700*	*dexB*	alpha-glucosidase	1.4	<0.01
SPD_0988	*SPD_RS05310*		YoaK family protein	1.4	0.03
SPD_1097	*SPD_RS05870*		CPBP family intramembrane metalloprotease	1.4	0.04
SPD_1788	*SPD_RS09520*		TatD family hydrolase	1.5	0.01
SPD_0814	*SPD_RS04365*		agmatine deiminase	1.5	0.01
SPD_0002	*SPD_RS00010*	*dnaN*	DNA polymerase III subunit beta	1.5	0.01

SPD_0074	*SPD_RS00395*		nucleoside phosphorylase	1.5	0.02
SPD_1465	*SPD_RS07790*		FtsX-like permease family protein	1.5	0.01
SPD_1878	*SPD_RS09965*		glutamyl-tRNA synthetase	1.5	0.01
SPD_1466	*SPD_RS07795*		ABC transporter ATP-binding protein	1.5	0.03
SPD_0926	*SPD_RS04990*		diacylglycerol kinase family lipid kinase	1.5	0.03
SPD_1445	*SPD_RS07685*		HAMP domain-containing histidine kinase	1.5	0.03
SPD_0833	*SPD_RS04470*	*trmFO*	methylenetetrahydrofolate--tRNA-(uracil(54)-C(5))-methyltransferase (FADH(2)-oxidizing) TrmFO	1.5	<0.01
SPD_0575	*SPD_RS03110*		sensor histidine kinase	1.5	0.01
SPD_0555	*SPD_RS02975*		ABC-2 family transporter protein	1.5	0.03
SPD_1131	*SPD_RS06035*	*carB*	carbamoyl-phosphate synthase large subunit	1.5	0.04
SPD_0437	*SPD_RS02350*		ECF transporter S component	1.5	<0.01
SPD_0178	*SPD_RS00990*		transcriptional regulator Spx	1.5	<0.01
SPD_1137	*SPD_RS06070*		ATP-binding cassette domain-containing protein	1.5	0.01
SPD_1205	*SPD_RS06440*	*aroA*	3-phosphoshikimate 1-carboxyvinyltransferase	1.5	<0.01
SPD_1449	*SPD_RS07710*		metallophosphoesterase family protein	1.5	0.01
SPD_0842	*SPD_RS04530*		GNAT family N-acetyltransferase	1.5	0.02
SPD_1923	*SPD_RS10185*	*dapD*	2,3,4,5-tetrahydropyridine-2,6-dicarboxylate N-acetyltransferase	1.5	<0.01
SPD_0260	*SPD_RS01415*	*rsuA-1*	pseudouridine synthase	1.5	<0.01
SPD_1448	*SPD_RS07705*		Rrf2 family transcriptional regulator	1.5	<0.01
SPD_1562	*SPD_RS08305*		mechanosensitive ion channel	1.5	<0.01
SPD_1115	*SPD_RS05950*	*leuB*	3-isopropylmalate dehydrogenase	1.5	0.01
SPD_1488	*SPD_RS07905*		ROK family protein	1.5	<0.01
SPD_1467	*SPD_RS07800*		glycoside hydrolase family 95 protein	1.5	<0.01
	SPD_RS11675		2-isopropylmalate synthase	1.5	0.03
SPD_0812	*SPD_RS04355*	*lys1*	saccharopine dehydrogenase family protein	1.6	<0.01
SPD_1821	*SPD_RS09695*	*pbp2A*	penicillin-binding protein PBP2A	1.6	<0.01
SPD_0646	*SPD_RS03475*		DegV family protein	1.6	0.01
SPD_1278	*SPD_RS06830*	*cppA*	CppA family protein	1.6	0.02
SPD_1665	*SPD_RS08880*	*treR*	trehalose operon repressor	1.6	0.02
SPD_1215	*SPD_RS06495*	*amy*	alpha-amylase	1.6	0.03
SPD_2007	*SPD_RS10610*		ryptide export MFS transporter	1.6	0.03
SPD_0616	*SPD_RS03315*		amino acid ABC transporter ATP-binding protein	1.6	0.02
SPD_1577	*SPD_RS08395*		type II toxin-antitoxin system HicB family antitoxin	1.6	0.01
SPD_1113	*SPD_RS05940*	*leuD*	3-isopropylmalate dehydratase small subunit	1.6	0.02
SPD_0885	*SPD_RS04760*	*ccdA-2*	thiol-disulfide oxidoreductase-associated membrane protein CcdA2	1.6	<0.01
SPD_0888	*SPD_RS04775*	*adcAII*	zinc-binding lipoprotein AdcAII	1.6	0.03
SPD_0813	*SPD_RS04360*	*nspC*	carboxynorspermidine decarboxylase	1.6	<0.01
SPD_0692	*SPD_RS03730*		hypothetical protein	1.6	<0.01
SPD_2028	*SPD_RS10730*	*cbpD*	choline binding-anchored murein hydrolase CbpD	1.6	0.04

SPD_0635	*SPD_RS03425*		heavy metal translocating P-type ATPase	1.6	<0.01
SPD_1414	*SPD_RS07520*		OFA family MFS transporter	1.6	<0.01
SPD_0475	*SPD_RS02565*		type II CAAX endopeptidase family protein	1.6	0.02
SPD_0782	*SPD_RS04180*		N-6 DNA methylase	1.6	0.02
SPD_1464	*SPD_RS07785*	*psaD*	thiol peroxidase	1.6	0.02
SPD_0624	*SPD_RS03365*	*thiE-1*	thiamine phosphate synthase	1.7	0.02
SPD_1118	*SPD_RS05965*	*cutC*	copper homeostasis protein CutC	1.7	<0.01
SPD_0811	*SPD_RS04350*	*speE*	polyamine aminopropyltransferase	1.7	<0.01
SPD_0538	*SPD_RS02885*	*uvrC*	excinuclease ABC subunit UvrC	1.7	<0.01
SPD_1028	*SPD_RS05525*	*acoA*	thiamine pyrophosphate-dependent dehydrogenase E1 component subunit alpha	1.7	<0.01
SPD_1138	*SPD_RS06075*	*htpX*	zinc metalloprotease HtpX	1.7	0.01
SPD_1910	*SPD_RS10125*	*pstS*	substrate-binding domain-containing protein	1.7	0.02
SPD_1985	*SPD_RS10505*		iron-containing alcohol dehydrogenase	1.7	0.03
SPD_1495	*SPD_RS07945*		ABC transporter substrate-binding protein	1.7	<0.01
SPD_0179	*SPD_RS00995*		SP0191 family lipoprotein	1.7	<0.01
SPD_0615	*SPD_RS03310*		transporter substrate-binding domain-containing protein	1.8	0.04
SPD_1680	*SPD_RS08955*	*birA*	bifunctional biotin--[acetyl-CoA-carboxylase] ligase/biotin operon repressor BirA	1.8	<0.01
SPD_1772	*SPD_RS09435*		acylphosphatase	1.8	0.03
SPD_1798	*SPD_RS09575*		response regulator transcription factor	1.8	<0.01
SPD_1678	*SPD_RS08945*	*aga*	alpha-galactosidase	1.8	<0.01
SPD_0357	*SPD_RS01940*	*cbpF*	choline-binding protein CbpF	1.8	<0.01
SPD_0220	*SPD_RS01210*		ACT domain-containing protein	1.8	0.02
SPD_1799	*SPD_RS09580*		sensor histidine kinase	1.8	<0.01
SPD_0685	*SPD_RS03695*	*gor*	glutathione-disulfide reductase	1.8	<0.01
SPD_1446	*SPD_RS07690*		response regulator transcription factor	1.8	<0.01
SPD_0632	*SPD_RS03410*	*thiD*	bifunctional hydroxymethylpyrimidine kinase/phosphomethylpyrimidine kinase	1.9	<0.01
SPD_1969	*SPD_RS10420*		beta-N-acetylhexosaminidase	1.9	<0.01
SPD_0809	*SPD_RS04340*	*cad*	aminotransferase class I/II-fold pyridoxal phosphate-dependent enzyme	1.9	<0.01
SPD_1637	*SPD_RS08730*		MerR family transcriptional regulator	1.9	0.03
SPD_1769	*SPD_RS09425*		Bax inhibitor-1/YccA family protein	1.9	<0.01
SPD_1005	*SPD_RS05390*	*glgB*	1,4-alpha-glucan branching protein GlgB	1.9	<0.01
SPD_1190	*SPD_RS06360*		TRZ/ATZ family protein	1.9	<0.01
SPD_0099	*SPD_RS00530*	*capD*	nucleoside-diphosphate sugar epimerase/dehydratase	1.9	<0.01
SPD_1773	*SPD_RS09440*	*yidC*	membrane protein insertase YidC	1.9	<0.01
SPD_1026	*SPD_RS05515*		dihydrolipoamide acetyltransferase	1.9	<0.01
SPD_0092	*SPD_RS00495*		DUF4299 family protein	1.9	<0.01
SPD_0185	*SPD_RS01025*	*cls*	cardiolipin synthase	1.9	<0.01
SPD_1592	*SPD_RS08495*		GNAT family N-acetyltransferase	1.9	0.01
SPD_2009	*SPD_RS10630*		hypothetical protein	2.0	<0.01

SPD_1122	*SPD_RS05990*	*dprA*	DNA-processing protein DprA	2.0	0.03
SPD_1246	*SPD_RS06655*	*nagB*	glucosamine-6-phosphate deaminase	2.0	<0.01
SPD_1800	*SPD_RS09585*		ABC transporter permease	2.0	<0.01
SPD_1119	*SPD_RS05970*		YbaN family protein	2.0	<0.01
SPD_1970	*SPD_RS10425*		ROK family protein	2.0	<0.01
SPD_0302	*SPD_RS01655*		hypothetical protein	2.0	<0.01
SPD_0889	*SPD_RS04780*	*phtD*	pneumococcal histidine triad protein PhtD	2.0	<0.01
SPD_0107	*SPD_RS00570*		bacteriocin-associated integral membrane family protein	2.0	<0.01
SPD_1989	*SPD_RS10525*		PTS system mannose/fructose/sorbose family transporter subunit IID	2.0	0.04
SPD_0066	*SPD_RS00335*		PTS system mannose/fructose/N-acetylgalactosamine-transporter subunit IIB	2.0	0.01
SPD_1027	*SPD_RS05520*		alpha-ketoacid dehydrogenase subunit beta	2.1	<0.01
SPD_0657	*SPD_RS03535*		CBS domain-containing protein	2.1	<0.01
SPD_0853	*SPD_RS04590*	*lytB*	endo-beta-N-acetylglucosaminidase	2.1	<0.01
SPD_0647	*SPD_RS03480*		TetR/AcrR family transcriptional regulator	2.1	<0.01
SPD_0844	*SPD_RS04540*	*celB*	DNA internalization-related competence protein ComEC/Rec2	2.1	<0.01
SPD_0914	*SPD_RS04910*	*rumA-1*	23S rRNA (uracil(1939)-C(5))-methyltransferase RlmD	2.1	<0.01
SPD_0927	*SPD_RS04995*	*nplT*	glycoside hydrolase family 13 protein	2.1	<0.01
SPD_0868	*SPD_RS04670*	*prsA*	peptidylprolyl isomerase PrsA	2.1	<0.01
SPD_1023	*SPD_RS05500*	*xerS*	tyrosine recombinase XerS	2.1	<0.01
SPD_0843	*SPD_RS04535*	*celA*	ComEA family DNA-binding protein	2.1	0.01
	SPD_RS05945		DUF1294 domain-containing protein	2.1	0.01
SPD_0851	*SPD_RS04580*	*pyrK*	dihydroorotate dehydrogenase electron transfer subunit	2.1	<0.01
SPD_1974	*SPD_RS10445*		alpha-L-fucosidase	2.1	<0.01
SPD_1447	*SPD_RS07695*		DUF2974 domain-containing protein	2.2	<0.01
SPD_0098	*SPD_RS00525*		glycosyltransferase family 2 protein	2.2	<0.01
SPD_1802	*SPD_RS09595*		hypothetical protein	2.2	0.02
SPD_1091	*SPD_RS05840*		ECF transporter S component	2.2	<0.01
SPD_1496	*SPD_RS07950*		PTS transporter subunit EIIC	2.2	<0.01
SPD_0351	*SPD_RS01905*		sensor histidine kinase	2.2	<0.01
	SPD_RS08490		transposase	2.2	<0.01
SPD_0852	*SPD_RS04585*	*pyrDb*	dihydroorotate dehydrogenase	2.2	<0.01
SPD_1679	*SPD_RS08950*	*msmR*	AraC family ligand binding domain-containing protein	2.2	<0.01
SPD_0352	*SPD_RS01910*		response regulator transcription factor	2.2	<0.01
SPD_0065	*SPD_RS00330*	*bgaC*	beta-galactosidase	2.3	<0.01
SPD_0289	*SPD_RS01590*	*eda*	bifunctional 4-hydroxy-2-oxoglutarate aldolase/2-dehydro-3-deoxy-phosphogluconate aldolase	2.3	<0.01
SPD_0803	*SPD_RS04305*		PspC domain-containing protein	2.3	<0.01
SPD_0609	*SPD_RS03280*	*pyrE*	orotate phosphoribosyltransferase	2.3	<0.01
SPD_0350	*SPD_RS01900*	*liaF*	cell wall-active antibiotics response protein LiaF	2.3	<0.01

SPD_0913	*SPD_RS04905*		DUF1002 domain-containing protein	2.3	<0.01
SPD_1996	*SPD_RS10560*		DeoR/GlpR family DNA-binding transcription regulator	2.3	<0.01
SPD_0247	*SPD_RS01345*	*bglA*	6-phospho-beta-glucosidase	2.3	<0.01
SPD_1172	*SPD_RS06255*	*nanE-2*	N-acetylmannosamine-6-phosphate 2-epimerase	2.4	<0.01
SPD_1025	*SPD_RS05510*	*lpdA*	dihydrolipoyl dehydrogenase	2.4	<0.01
SPD_0617	*SPD_RS03320*		amino acid ABC transporter permease	2.4	<0.01
SPD_1945	*SPD_RS10305*		DUF3169 family protein	2.4	<0.01
SPD_1636	*SPD_RS08725*		alcohol dehydrogenase catalytic domain-containing protein	2.4	<0.01
SPD_1504	*SPD_RS07990*	*nanA*	LPXTG-anchored neuraminidase NanA	2.5	<0.01
SPD_0023	*SPD_RS00110*	*comW*	sigma(X)-activator ComW	2.5	0.02
SPD_1801	*SPD_RS09590*		ABC transporter ATP-binding protein	2.5	<0.01
SPD_2005	*SPD_RS10600*	*dltA*	D-alanine--poly(phosphoribitol) ligase subunit DltA	2.5	<0.01
SPD_1702	*SPD_RS09065*	*rrsB*	16S ribosomal RNA	2.5	<0.01
SPD_0016	*SPD_RS00075*	*rrsA*	16S ribosomal RNA	2.5	<0.01
SPD_1893	*SPD_RS10040*	*rrsD*	16S ribosomal RNA	2.5	<0.01
SPD_1816	*SPD_RS09665*	*rrsC*	16S ribosomal RNA	2.5	<0.01
SPD_1990	*SPD_RS10530*		PTS mannose/fructose/sorbose/N-acetylgalactosamine transporter subunit IIC	2.5	0.01
SPD_0297	*SPD_RS01630*		PTS system mannose/fructose/sorbose family transporter subunit IID	2.5	<0.01
SPD_2002	*SPD_RS10585*	*dltD*	D-alanyl-lipoteichoic acid biosynthesis protein DltD	2.5	<0.01
SPD_0444	*SPD_RS02385*		bacterial Ig-like domain-containing protein	2.5	<0.01
SPD_0717	*SPD_RS03860*	*clpE*	ATP-dependent Clp protease ATP-binding subunit	2.6	<0.01
SPD_0804	*SPD_RS04315*		ABC transporter ATP-binding protein	2.6	<0.01
SPD_1375	*SPD_RS07310*		NADPH-dependent FMN reductase	2.6	<0.01
SPD_1582	*SPD_RS08435*		glycoside hydrolase family 32 protein	2.6	<0.01
SPD_2004	*SPD_RS10595*	*dltB*	D-alanyl-lipoteichoic acid biosynthesis protein DltB	2.6	<0.01
SPD_1024	*SPD_RS05505*		lipoate--protein ligase	2.6	<0.01
SPD_1971	*SPD_RS10430*		alpha-mannosidase	2.7	<0.01
SPD_0249	*SPD_RS01355*		LLM class flavin-dependent oxidoreductase	2.8	<0.01
SPD_1994	*SPD_RS10550*	*fucA*	L-fuculose-phosphate aldolase	2.8	<0.01
SPD_0890	*SPD_RS04785*	*phtE*	pneumococcal histidine triad protein PhtE	2.8	<0.01
SPD_2035	*SPD_RS10765*		DEAD/DEAH box helicase	2.8	0.01
SPD_1531	*SPD_RS08145*	*scrK*	ROK family protein	2.8	<0.01
SPD_0303	*SPD_RS01660*		helix-turn-helix transcriptional regulator	2.8	<0.01
SPD_1944	*SPD_RS10300*		type II CAAX endopeptidase family protein	2.9	<0.01
SPD_0294	*SPD_RS01615*		glycoside hydrolase family 88 protein	2.9	<0.01
SPD_1037	*SPD_RS05570*		pneumococcal-type histidine triad protein	2.9	<0.01
SPD_0608	*SPD_RS03275*	*pyrF*	'orotidine-5''-phosphate decarboxylase'	2.9	<0.01
SPD_0290	*SPD_RS01595*		PfkB family carbohydrate kinase	2.9	<0.01

SPD_0559	*SPD_RS03015*		PTS sugar transporter subunit IIA	2.9	<0.01
SPD_2003	*SPD_RS10590*	*dltC*	D-alanine--poly(phosphoribitol) ligase subunit DltC	3.0	<0.01
SPD_0360	*SPD_RS01950*	*mtlA*	PTS mannitol-specific transporter subunit IIBC	3.0	<0.01
SPD_0805	*SPD_RS04320*		ABC transporter permease	3.0	<0.01
SPD_0068	*SPD_RS00345*		PTS system mannose/fructose/sorbose family transporter subunit IID	3.0	<0.01
SPD_0610	*SPD_RS11315*		hypothetical protein	3.0	<0.01
SPD_1710	*SPD_RS09105*	*groES*	co-chaperone GroES	3.0	<0.01
SPD_1709	*SPD_RS09100*	*groL*	chaperonin GroEL	3.0	<0.01
SPD_0618	*SPD_RS03325*		amino acid ABC transporter permease	3.1	<0.01
SPD_1057	*SPD_RS05665*		PTS sugar transporter subunit IIB	3.1	0.01
SPD_2069	*SPD_RS10950*		ParB/RepB/Spo0J family partition protein	3.1	<0.01
SPD_1497	*SPD_RS07955*	*nanE-1*	N-acetylmannosamine-6-phosphate 2-epimerase	3.1	<0.01
SPD_2068	*SPD_RS10945*		trypsin-like peptidase domain-containing protein	3.2	<0.01
SPD_1053	*SPD_RS05650*	*lacA*	galactose-6-phosphate isomerase subunit LacA	3.3	<0.01
SPD_1046	*SPD_RS05615*	*lacG-2*	6-phospho-beta-galactosidase	3.3	<0.01
SPD_0071	*SPD_RS00360*	*galM*	galactose mutarotase	3.3	<0.01
SPD_1047	*SPD_RS05620*	*lacE-2*	lactose-specific PTS transporter subunit EIIC	3.3	<0.01
SPD_0291	*SPD_RS01600*		RpiB/LacA/LacB family sugar-phosphate isomerase	3.3	<0.01
SPD_0069	*SPD_RS00350*		PTS sugar transporter subunit IIA	3.4	<0.01
SPD_1946	*SPD_RS10310*		hypothetical protein	3.4	<0.01
SPD_1972	*SPD_RS10435*		glycoside hydrolase family 125 protein	3.4	<0.01
SPD_1267	*SPD_RS06770*		ABC transporter ATP-binding protein	3.4	<0.01
SPD_0457	*SPD_RS02460*		DUF2812 domain-containing protein	3.5	<0.01
SPD_1891	*SPD_RS10030*	*rrlD*	23S ribosomal RNA	3.5	<0.01
SPD_0018	*SPD_RS00085*	*rrlA*	23S ribosomal RNA	3.5	<0.01
SPD_1700	*SPD_RS09055*	*rrlB*	23S ribosomal RNA	3.5	<0.01
SPD_1814	*SPD_RS09655*	*rrlC*	23S ribosomal RNA	3.5	<0.01
SPD_1052	*SPD_RS05645*	*lacB*	galactose-6-phosphate isomerase subunit LacB	3.5	<0.01
SPD_0461	*SPD_RS02485*	*dnaJ*	molecular chaperone DnaJ	3.6	<0.01
SPD_1050	*SPD_RS05635*	*lacD*	tagatose-bisphosphate aldolase	3.6	<0.01
SPD_1535	*SPD_RS08170*	*scrR*	LacI family DNA-binding transcriptional regulator	3.7	<0.01
SPD_0701	*SPD_RS03775*	*ciaR*	two-component system response regulator CiaR	3.7	<0.01
SPD_0067	*SPD_RS00340*		PTS mannose/fructose/sorbose/N-acetylgalactosamine transporter subunit IIC	3.8	<0.01
SPD_1948	*SPD_RS10320*		hypothetical protein	3.8	<0.01
SPD_0070	*SPD_RS00355*	*agaS*	SIS domain-containing protein	3.8	<0.01
SPD_0865	*SPD_RS04655*	*coiA*	competence protein CoiA	3.8	<0.01
SPD_0292	*SPD_RS01605*		gluconate 5-dehydrogenase	3.8	<0.01
	SPD_RS02480		hypothetical protein	3.8	<0.01
SPD_0702	*SPD_RS03780*	*ciaH*	two-component system sensor histidine kinase CiaH	3.9	<0.01
SPD_1718	*SPD_RS09145*		LytTR family DNA-binding domain-containing	3.9	<0.01

			protein		
SPD_0072	*SPD_RS00365*		VOC family protein	3.9	<0.01
SPD_1534	*SPD_RS08165*	*scrB*	sucrose-6-phosphate hydrolase	3.9	<0.01
SPD_1947	*SPD_RS10315*		helix-turn-helix transcriptional regulator	4.2	<0.01
SPD_1932	*SPD_RS10235*	*malP*	glycogen/starch/alpha-glucan family phosphorylase	4.2	<0.01
SPD_1051	*SPD_RS05640*	*lacC*	tagatose-6-phosphate kinase	4.2	<0.01
SPD_1503	*SPD_RS07985*		YhcH/YjgK/YiaL family protein	4.3	<0.01
SPD_1717	*SPD_RS09140*		membrane protein	4.5	<0.01
SPD_0625	*SPD_RS03375*		ECF transporter S component	4.5	<0.01
SPD_1933	*SPD_RS10240*	*malQ*	4-alpha-glucanotransferase	4.7	<0.01
SPD_0560	*SPD_RS03020*		PTS sugar transporter subunit IIB	4.7	<0.01
SPD_2010	*SPD_RS10635*		hypothetical protein	4.8	<0.01
SPD_2034	*SPD_RS10760*		ComF family protein	4.8	0.02
SPD_0460	*SPD_RS02475*	*dnaK*	molecular chaperone DnaK	4.9	<0.01
SPD_1498	*SPD_RS07960*		Gfo/Idh/MocA family oxidoreductase	5.0	<0.01
SPD_1506	*SPD_RS08000*		acetylxylan esterase	5.2	<0.01
SPD_1995	*SPD_RS10555*	*fucK*	rhamnulokinase family protein	5.3	<0.01
SPD_0628	*SPD_RS03390*	*tenA*	thiaminase II	5.3	<0.01
SPD_0090	*SPD_RS00485*		ABC transporter substrate-binding protein	5.5	<0.01
SPD_0627	*SPD_RS03385*		energy-coupling factor transporter transmembrane component T	5.7	<0.01
SPD_1532	*SPD_RS08150*		sucrose-specific PTS transporter subunit IIBC	5.9	<0.01
SPD_0626	*SPD_RS03380*		ABC transporter ATP-binding protein	5.9	<0.01
SPD_0775	*SPD_RS04150*		hypothetical protein	5.9	<0.01
SPD_0629	*SPD_RS03395*	*thiW*	energy coupling factor transporter S component ThiW	6.0	<0.01
SPD_1716	*SPD_RS09135*		cell wall-binding protein	6.1	<0.01
SPD_0458	*SPD_RS02465*	*hrcA*	heat-inducible transcriptional repressor HrcA	6.1	<0.01
SPD_0459	*SPD_RS02470*	*grpE*	nucleotide exchange factor GrpE	6.6	<0.01
SPD_0106	*SPD_RS00565*		lactococcin 972 family bacteriocin	6.6	<0.01
SPD_0631	*SPD_RS03405*	*thiE-2*	thiamine phosphate synthase	6.7	<0.01
SPD_0562	*SPD_RS03035*	*bgaA*	LPXTG-anchored adhesin/beta-galactosidase BgaA	6.9	<0.01
SPD_0089	*SPD_RS00475*		carbohydrate ABC transporter permease	6.9	<0.01
SPD_0561	*SPD_RS03025*		PTS galactitol transporter subunit IIC	7.2	<0.01
SPD_0630	*SPD_RS03400*		hydroxyethylthiazole kinase	7.3	<0.01
SPD_1499	*SPD_RS07965*	*nanB*	neuraminidase NanB	7.5	<0.01
	SPD_RS11940		hypothetical protein	7.7	<0.01
SPD_0088	*SPD_RS00470*		sugar ABC transporter permease	7.8	<0.01
SPD_1505	*SPD_RS07995*		hypothetical protein	8.2	<0.01
SPD_0162	*SPD_RS00905*		DUF3021 domain-containing protein	8.2	<0.01
SPD_1977	*SPD_RS10465*	*arcC*	carbamate kinase	8.4	<0.01
SPD_2013	*SPD_RS10650*	*glpK*	glycerol kinase GlpK	8.4	<0.01
SPD_2011	*SPD_RS10640*	*glpF*	aquaporin family protein	8.6	<0.01

SPD_1978	*SPD_RS10470*		YfcC family protein	8.8	<0.01
SPD_0163	*SPD_RS00910*		LytTR family DNA-binding domain-containing protein	9.2	<0.01
SPD_1501	*SPD_RS07975*		sugar ABC transporter permease	9.6	<0.01
SPD_1502	*SPD_RS07980*		sugar ABC transporter substrate-binding protein	10.7	<0.01
SPD_2012	*SPD_RS10645*	*glpO*	type 1 glycerol-3-phosphate oxidase	11.3	<0.01
SPD_1500	*SPD_RS07970*		carbohydrate ABC transporter permease	12.0	<0.01
SPD_1979	*SPD_RS10475*		dipeptidase	12.0	<0.01
SPD_0080	*SPD_RS00430*	*pavB*	fibronectin-binding SSURE repeat adhesin PavB	15.6	<0.01
SPD_1976	*SPD_RS10460*	*argF*	ornithine carbamoyltransferase	16.7	<0.01
SPD_0308	*SPD_RS01685*	*clpL*	ATP-dependent Clp protease ATP-binding subunit	19.6	<0.01
	SPD_RS10900		YhgE/Pip family protein	25.7	<0.01
	SPD_RS11410		hypothetical protein	27.5	<0.01
	SPD_RS11405		YhgE/Pip family protein	27.7	<0.01
